# *Bacillus subtilis* Produces Amino Acids to Stimulate Protein Synthesis in Ruminal Tissue Explants *via* the Phosphatidylinositol-4,5-Bisphosphate 3-Kinase Catalytic Subunit Beta–Serine/Threonine Kinase–Mammalian Target of Rapamycin Complex 1 Pathway

**DOI:** 10.3389/fvets.2022.852321

**Published:** 2022-06-27

**Authors:** Qiuju Wang, Yulong Ren, Yizhe Cui, Bingnan Gao, Hao Zhang, Qianming Jiang, Juan J. Loor, Zhaoju Deng, Chuang Xu

**Affiliations:** ^1^College of Animal Science and Veterinary, Heilongjiang Bayi Agricultural University, Daqing, China; ^2^Department of Animal Sciences and Division of Nutritional Sciences, University of Illinois, Urbana, IL, United States; ^3^College of Veterinary Medicine, China Agricultural University, Beijing, China

**Keywords:** *Bacillus subtilis*, rumen explants, protein expression, amino acids, mTOR

## Abstract

**Background:**

*Bacillus subtilis* is a probiotic strain that is widely used as a feed supplement for ruminants. In this study, one *B. subtilis* strain isolated from the ruminal fluid of Holstein dairy cows was used for an *ex vivo* study with ruminal tissue explants. The main goal was to assess the potential endosymbiotic links between *B. subtilis* and the ruminal epithelium using molecular analyses and amino acid profiling. The explant culture protocol was first optimized to determine the ideal conditions in terms of tissue viability before performing the actual experiments involving active and inactive bacteria with or without protein synthesis inhibitors, such as LY294002 (phosphatidylinositol 3-kinase inhibitor) or rapamycin [mammalian target of rapamycin (mTOR) inhibitor].

**Results:**

The mRNA levels of phosphatidylinositol-4,5-bisphosphate 3-kinase catalytic subunit beta (*PIK3CB*), serine/threonine kinase (*AKT*), *mTOR, P70S6K1*, and eukaryotic translation initiation factor 4E binding protein 1 were the highest (*p* < 0.01), while those of programmed cell death 4 were the lowest when the tissue was incubated with 10^7^ of *B. subtilis*. Compared with the inactivated bacteria, the expression levels of *PIK3CB* and *AKT*, and overall changes in *mTOR* and *P70S6K1* were greater in rumen explants with living bacteria (*p* < 0.05). With an increase in *B. subtilis* concentration, the trends of protein and corresponding gene changes were consistent. There were differences in the concentrations of individual amino acids in the supernatants of living and inactivated bacterial culture groups, with most amino acids enriched in pathways, such as aminoacyl tRNA biosynthesis, cyanoamino acid metabolism, monobactam biosynthesis, or glycine, serine, and threonine metabolism. The addition of psilocybin upregulated the expression levels of *PIK3CB* and *AKT*. A significant decrease (*p* < 0.05) in PIK3CB and mTOR protein expression levels was detected after the addition of LY294002 and rapamycin. In addition, These responses were associated with the downregulation (*p* < 0.05) of AKT and P70S6K protein expression levels.

**Conclusions:**

We confirmed that the *in vivo* ruminal tissue culture system is a suitable model for studying probiotic-induced alterations in tissue function. As such, this study provides a means for future mechanistic studies related to microbial regulation and the dietary supply of proteins. In addition, living and inactivated *B. subtilis* can promote protein synthesis in ruminal tissue explants by altering the expression levels of related factors in the PIK3CB–AKT–mTORC1 pathway, which could further aid in optimizing the feed efficiency and increasing the use of inactivated bacteria as additives in dairy cow farming.

## Introduction

*Bacillus subtilis* is a non-pathogenic transitional microorganism of the digestive tract (1). When fed to cows, *B. subtilis* can improve ruminal fermentation and produce various enzymes to promote digestion and absorption in the intestinal tract (2). *Bacillus subtilis* not only breaks down ratio proteins and circulates urea back to the rumen, but also promotes the absorption of amino acids and peptides produced by decomposition, promotes protein synthesis, and increases the number of proteins reaching the intestine (3–5). *Bacillus subtilis* has been a safe-to-use probiotic for a long time. In recent years, many researchers have studied the effects of *B. subtilis* on ruminants, including calves and lactating cows, and found that can potentially be used as a probiotic for dairy cows (6). However, the specific mechanism of action of *B. subtilis* remains unclear, mainly because the addition of *B. subtilis* is generally in the form of liquid or solid fermentation cultures containing not only bacteria but also components of the culture medium (7).

The physiology and metabolism of microbial cultures fed to ruminants can be studied through biochemical characteristics or specific phenotype analyses (8, 9), especially aspects related to the synthesis and metabolism of nutrients in the rumen (10). Dietary protein sources in the feed are costly (11), and identifying microorganisms that can promote protein synthesis could help to improve the feed utilization efficiency in dairy cows and increase the protein content in milk. The promotion of rumen protein synthesis not only effectively increases nitrogen retention in ruminants, but also reduces the emission of nitrogenous compounds (12) and wastage of protein feed resources, while improving the production performance of ruminants (13).

PIK3CB–AKT–mTOR1 is a critical pathway that controls protein synthesis in the tissues. Mammalian target of rapamycin complex 1 (mTORC1) can be activated by receiving signals from amino acids, growth factors, and nutrients, ultimately leading to the activation of downstream factors that regulate a variety of metabolic activities, including protein synthesis and degradation (14). When phosphatidylinositol-4,5-bisphosphate 3-kinase catalytic subunit beta (PIK3CB), phosphorylated mammalian target of rapamycin (p-mTOR), and phosphorylated programmed cell death protein 4 (p-PDCD4) are activated, phosphorylated serine/threonine kinase (p-AKT) begins to promote phosphorylation of mTORC1, which can activate transcription and translation by phosphorylating eukaryotic translation initiation factor 4E binding protein 1 (p-4EBP1) and phosphorylated ribosomal protein S6 kinase 1(p-P70S6K). Phosphatidylinositol 3-kinase (PI3K) is a member of the intracellular phosphatidylinositol kinase class, and many downstream signaling pathways are involved in the regulation of translation initiation (15). When PI3K is activated, it is transferred from the cytosol to the cell membrane, activating AKT and phosphorylating its Thr308 residue. In turn, activation of AKT phosphorylates many of its downstream effectors, including mTOR. mTOR is an important regulator of protein synthesis and one of the most important downstream substrates of AKT (16). Ribosomal S6 kinase (S6K) and elF4E binding protein (4EBP1) are the two most common substrates of mTORC1. Phosphorylation of 4EBP1 and S6K is dependent on the Tor signal transduction sequence (TOS) motif, which binds to Raptor and is present in the negative regulator PRAS40, which promotes protein synthesis (17). mTOR is more sensitive to Met, Ile, Thr, and Leu and can transduce synthetic signals to a larger protein synthesis process (18). Burgos showed that when all amino acids were removed from the medium, the phosphorylation levels of many substrates of mTORC1 were significantly reduced, thereby slowing down the protein synthesis process (19).

In addition, Cadherin 1 (*CDH1*) is an important marker of intercellular adhesion and epithelial cell differentiation and plays a more important role in maintaining the structural integrity and metabolism of rumen epithelial cells than N-cadherin and epidermal growth factor receptor. We had previously detected the expression of *CDH1* by assaying the total RNA extracted from rumen explants (20, 21). *In vitro* cell culture models have been developed and used to reduce the use of experimental animals. However, cultured monolayers of cells differ significantly from the complex three-dimensional tissue structure and have various drawbacks. *In vitro* explant culture is not only an alternative to *in vivo* models, but it also retains histological features similar to those *in vivo* in a controlled environment. Even though the tissues used are still harvested from animals, the number of individual animals can be greatly reduced using this modeling approach.

Compared to cell culture, explant cultures have more advantages. Some types of cells are difficult to culture (22), but explant culture technology can overcome this limitation to some extent as it is not necessary for the cultured tissue cells to have cell lines. Furthermore, the rumen explants of young calves are more active and stable than those of mature cows, and they do not need to be sub-generated as many times as cells, and the experiment can be started just after the explants are stable (23). Moreover, the rumen explant culture time is relatively flexible, and according to the specific experimental needs, the explants can also be used to extract tissue proteins and RNA for subsequent experiments (24).

In the present study, an *in vitro* explant culture model of Holstein dairy cows was established to investigate whether the inactivation or activation of *B. subtilis* affected amino acid production and protein synthesis during rumen explant culture. In previous studies, *B. subtilis* was found to be highly tolerant to both acids and bases, to be stable in the gastrointestinal tract of cows, and secrete substances related to protein metabolism because of its ability to grow in the rumen (6, 25). Compared with living bacteria, inactivated bacteria are safer to use, avoiding the risk of mutation, abnormal site transfer, and a possible increase in drug resistance gene transfer in living bacteria. The stability of inactivated bacteria is higher, it does not need to maintain a certain number of living bacteria, it is easier to transport and preserve, and the quality and stability are also higher. In addition, inactivated bacteria can be combined with other additives, such as antibiotics and butyrate, whereas living bacteria are susceptible to sensitivity reactions when used with other additives. Therefore, we hypothesized that *B. subtilis* living and heat-inactivated bacteria can promote rumen explant protein synthesis *via* the PI3K–AKT–mTORC1 pathway. We aimed to check whether inactivated bacteria could perform similar functions as living bacteria and to provide an experimental basis for the use of probiotic non-essential living bacteria.

## Materials and Methods

### *B. subtilis* IIVE-4 Isolation and Identification

Ten high-yielding Holstein dairy cows (695.2 ± 33.5 kg) from a dairy farm in Jiusan (Heilongjiang Province, China) were selected for ruminal fluid collection using a nasopharyngeal tube before morning feeding. After bottling, the vessels were wrapped with a sealing membrane and quickly transported to the laboratory in an insulation foam box. Sterile medical gauze was folded into four layers, poured into the rumen fluid, and filtered. The filter was then placed in a new sterile blue cap bottle and centrifuged at 100 × *g* for 10 min. The supernatant of rumen fluid from all cows was spread on tryptose soy agar (TSA) plates (Qingdao Hope Bio-Technology Co. Ltd., Qingdao City, China) to isolate the bacteria. All strains grown on TSA plates for 24 h at 37°C under anaerobic and static conditions were cultured by repeating this step 4–5 times to obtain the purified strain. *Bacillus subtilis* was initially isolated from rumen fluid samples, and bacterial DNA was extracted using the Tian Gen kit and amplified by polymerase chain reaction (PCR). Amplified products were sent to Shanghai Bioengineering Co., Ltd. for sequencing.

The obtained bacterial solution was diluted with saline, and 100 μl of the diluted bacterial solution was extracted and evenly coated onto TSA for 24 h at 37°C. The number of colonies on the plate was counted and the concentration of the original bacterial solution was calculated and diluted to different concentrations (10^4^, 10^5^, 10^6^, 10^7^,10^8^, and 10^9^ CFU/ml) using Luria–Bertani (LB) broth for the rumen explant assay. The inactivated bacterial broth was obtained by placing the living bacteria broth in a sterilizer at 121°C for 15 min. The bacterial culture supernatant was collected by centrifugation (100 × *g* at 4°C for 10 min). The biological characteristics of the target bacteria were determined. The tolerance of the bacterial solution under different culture conditions was determined. Different temperatures at 32, 34, 36, 37, 38, and 40°C; different pH at 6.0, 6.5, 7.0, 7.5, 8.0; different bile salt concentrations of 0.5, 1.0, 1.5, and 2.0%; all cultures were incubated in a shaking table at 150 revolutions per min (rpm/min) for 24 h, and absorbance was determined at 600 nm using a microplate reader.

### Collection of Rumen Explants and Screening of Culture Conditions

The Ethics Committee of Heilongjiang Bayi Agricultural University (Daqing, China) approved the study protocol for the use and care of animals. Animal studies were performed in accordance with the guiding principles adopted by the Chinese Association for Laboratory Animal Sciences. Three lactating Holstein cows were selected from a farm slaughterhouse in Daqing, Heilongjiang province. Average parity, body weight, days in milk, and milk yield prior to slaughter of the cows were 3.6 ± 0.6, 686 ± 25.2, 228.6 ± 22.2, and 30.3 ± 2.1 kg/day [mean ± standard deviation (SD)], respectively. The collection and culture of rumen explants were carried out according to the method described by Zhang et al. (20) and Fathi et al. (26). Briefly, the rumen from each cow was emptied and surface debris was rinsed off with saline. A subsample of tissue was placed in a beaker containing a mixture of phosphate buffer saline (PBS, contained 2.5 mg/ml amphotericin B, 100 mg/ml streptomycin, 50 mg/ml penicillin, and 50 mg/ml gentamicin, pH 7.4, 37°C). After rinsing the rumen tissue with PBS to remove residual feed, the rumen mucosal layer was separated and discarded using sterile forceps. The mucosal tissue was cut into 4 mm^2^ sections with a surgical scalpel and incubated in duplicate with the medium. The explant surface was rinsed several times with PBS to wash off the blood and other debris. During the isolation process, tissue from three cows was washed several times with PBS at 37°C to slow-down tissue activity. Three separate tissue blocks from each cow were then laid out nipple-up with sterile forceps in 24-well plates containing 2.5 mL of culture medium A or B and incubated at 37°C in a 5% CO_2_ incubator.

To detect the activity of rumen explants at different times, explants from 0, 2, 8, 12, 16, 20, 24, 48, and 72 h were rinsed 4–5 times with the PBS mixture and placed in culture plates with 2 ml of medium containing 3-(4,5-dimethylthiazol-2-yl)-2,5-diphenyl tetrazolium bromide (MTT) components and incubated at 37°C with 5% CO_2_ for 4 h. Blue-purple methanamine (formazan) was allowed to use in tissue cells to be fully deposited. At the end of the incubation, the MTT-containing medium on the surface of the explants was washed off with PBS and immersed in a methanol solution to allow complete precipitation of methane. The explants were removed and placed in a drying oven for 30 min, the dry weight of the explants was recorded, and the optical density of the solution containing methanate at 570 nm was measured using an enzyme marker to calculate the explant viability. The formula used was explant viability = optical density/explant dry weight × 100% (20).

To evaluate the optimal culture time and medium for subsequent experiments, two series of cultures were used. First, medium A contained 80% Dulbecco's modified eagle medium: nutrient mixture F-12 (DMEM/F12 medium), 4% insulin, transferrin, and selenium additive, 100 mg/ml penicillin, 2.5 mg/ml amphotericin B, 100 mg/ml streptomycin, 50 mg/ml gentamicin, 2 mM/L glutamine, and 10% heat-inactivated serum. The second series involved using medium B containing 90% DMEM/F12 medium, 4% insulin, transferrin, and selenium additive (100 ×), 100 mg/ml penicillin, 2.5 mg/ml amphotericin B, 100 mg/mL streptomycin, 50 mg/ml gentamicin, and 2 mM/ml L-glutamine. The culture time encompassed 10-time points from 0 to 72 h (0, 2, 4, 8, 12, 16, 20, 24, 48, and 72 h), and samples from each culture series were collected at each time point to assess the activity of the explants. After sample collection, a portion was fixed in 4% formaldehyde, paraffin-embedded, sectioned and subjected to hematoxylin and eosin (HE) staining to observe structural integrity under a microscope. The remaining portion of each sample was frozen in liquid nitrogen. Subsequently, the tissue was ground and crushed, and TRIzol reagent (Catalog No. 15596-018, LIFE) was added to extract the RNA. After reverse transcription, *CDH1* gene expression levels in the explants were measured using quantitative fluorescence PCR. The reaction conditions were 95.0°C for 3 min, 95.0°C for 10 s, 56.0°C for 30 s, 72.0°C for 45 s, and melting for 15 s over 40 cycles.

### Stimulation of Ruminal Explants With *B. subtilis* and Its Effects on RNA and Protein Expression

Freshly-isolated explants from each of the three cows were incubated in triplicates for 1 h at 37°C with 5% CO_2_. Briefly, explants were removed and rinsed 4 to 5 times with PBS at 37°C, and then were placed in a new 24-well plate containing 1.8 ml of medium A. Then, 200 μl of 2.0 × 10^4^, 10^5^, 10^6^, 10^7^, 10^8^, and 10^9^ CFU/ml of living and inactivated *B. subtilis*. The bacterial liquid was placed in an autoclave at 121°C for 30 min, prolonged high temperatures could denature the proteins in bacteria, causing them to lose their biological role. After that, the inactivated bacterial solution was coated on the nutrient agar medium and was cultured at 37°C for 24 h. If no colonies were generated, the inactivated bacterial solution was used for subsequent tests. About 200 μl of *B. subtilis* solution was added to the culture plates containing rumen explants and 1.8 ml of medium. After 2 h of stimulation, all liquid in the plates was discarded, and the rumen explants were rinsed with PBS 4 to 4–5 times and placed into new plates with 2.5 ml of medium A in each well. Incubation was continued in a CO_2_ incubator at 37°C with 5% CO_2_ for 12 h. LB broth was used as a control group.

Total RNA was extracted from rumen explants and reverse-transcribed into cDNA using an RNAiso Plus kit (TaKaRa Biotechnology Co. Ltd., Beijing, China) according to the manufacturer's protocol. Subsequently, a reverse transcription kit (RR047A; TaKaRa Biotechnology Co., Ltd., Beijing, China) was used to synthesize cDNA with 1 μg of total RNA according to the supplier's protocols. The SYBR Green Plus Reagent Kit (Roche, Shanghai, China) was used for qRT-PCR assays with a 7500 Real-Time PCR System (Applied Biosystems). Six key factors in the PIK3CB–AKT–mTORC1 pathway, *4EBP1, AKT, MTOR, P70S6K, PIK3CB*, and*-PDCD*, were selected as target genes for qPCR (Applied Biosystems, Analytik Jena, Germany). Raw data were analyzed using the comparative quantification method, and the 2^−ΔΔCt^ method was used to calculate the relative expression of the target genes, with actin serving as an internal control. Primers were designed using Primer Premier 5 Tool (Premier; Supplementary Table 1; https://doi.org/10.6084/m9.figshare.14660184.v1). The reaction conditions for fluorescence quantitative PCR were: 95.0°C for 3 min, 95.0°C for 10 s, 56.0°C for 30 s, 72.0°C for 45 s, and melting for 15 s over 40 cycles. All samples were analyzed in triplicate.

Western blotting was performed to determine the abundance of six target proteins in the PIK3CB–AKT–mTORC1 pathway in rumen explants cultured at 37°C for 12 h. Total tissue proteins were extracted using a protein extraction kit (C510003; Sangon Biotech Co.Ltd., Shanghai, China) according to the manufacturer's instructions. The protein concentration was measured using the Enhanced BCA Protein Assay Kit (P0010, Beyotime Co. Ltd., Shanghai, China). Protein samples were denatured for 5 min at 100°C, separated on an 8% SDS-PAGE gel (30 μg of protein per sample), and electro-transferred onto polyvinylidene difluoride (PVDF) membranes (Bio-Rad, Shanghai, China). After blocking with blocking buffer Tris Buffered saline with Tween-20 (TBST) (Tris 50 mM for pH 7.6, NaCl 150 mM, and Tween-20 0.1%) containing 5% BSA (4240, Saiguo Biotech Co. Ltd., Guangzhou, China) for 2 h at room temperature, the membranes were then incubated with primary antibodies against PIK3CB (1:1,000, Ab40776, Abcam), PDCD4 (1:1,000, 9535S, Cell Signaling), site-specific phosphorylated AKT (Ser^473^, 1:1,000, 4060S, Cell Signaling), 4EBP1 (Thr^37^, 1:1,000, YP001, Immunoway), P70S6K (Thr^389^, 1:1000, YP1427, Immunoway), mTOR (Ser^2448^,1:2,000, YP0176, Immunoway), and β-Actin (1:10,000, 3700S, Cell Signaling Technology) overnight at 4°C.

The membranes were rinsed with TBST 3 times (10 min each time) and incubated with HRP-conjugated secondary antibodies (anti-mouse IgG1, 96714S, Cell Signaling; anti-rabbit IgG, 14708S, Cell Signaling) diluted at 3:5,000 in TBST for 30 min at room temperature. After brief washing, an enhanced chemiluminescence solution detection kit (ECL, Millipore) was used to visualize the specific bands. Image J2x 2.1.4.7 Analyzer (Rawak Software Inc., Stuttgart, Germany) was used to quantify the intensity of each band. Refer to (27–29); LY294002 (50 μmol/L) and rapamycin (10 nmol/L; 9904) levels were determined with reference to previous studies and combined with the results of previous toxicity tests (30).

After stable incubation of rumen explants for 1 h, LY294002 (50 μmol/L; 9901; Cell Signaling Technology) and rapamycin (10 nmol/L; 9904; Cell Signaling Technology) was added for 30 min, after which explants were removed and rinsed 4–5 times with PBS solution. Subsequently, they were stimulated with 2.0 × 10^7^ CFU/ml of living or inactivated bacteria for 12 h. Subsequently, the explants were washed three times before RNA extraction. Rumen explants treated with inhibitors served as the control group. As in the previous experiment, protein and RNA were extracted from each group of explants, and the tissue culture supernatant was collected.

### Determination of Free Amino Acids in the Supernatant

An automatic amino acid analyzer s-433d (Sykam, Germany) and a vacuum tube concentrator TVE-1100 (EYELA, Tokyo) was used for amino acid profiling. Hierarchical clustering analysis was performed on all significantly different free amino acids, and the results are reported in a heat map. Correlation analysis was then used to measure the closeness of the relationship between the statistically significant amino acids. The degree of linear correlation between two free amino acids was measured using the Pearson correlation coefficient, and the free amino acids with significantly different contents between groups were selected for visualization. Lastly, pathway enrichment analysis of significant free amino acids was performed using the Kyoto Encyclopedia of Genes and Genomes database for a better appreciation of the pathways contributing to amino acid and protein metabolism.

### Statistical Analysis

Data were analyzed using SPSS (version 22.0; SPSS Inc., Chicago, IL, USA) and GraphPad Prism 7.00 (GraphPadSoftware, San Diego, CA, USA). Results are expressed as the mean ± SD. All experiments were independently repeated at least three times. Statistical significance was determined using a two-tailed unpaired Student's *t*-test or one-way ANOVA followed by Tukey's *post-hoc* test for comparisons between two or more two groups, respectively. Differences were considered significant at ^*^p < 0.05 and highly-significant at ^**^p < 0.01. For the screening test of optimal culture conditions of explants (experiment II), differences in *CDH1* expression were used (“^*^” indicates a significant difference compared to the 0 h group, *p* < 0.05. “^**^” indicates highly significant difference compared to 0 h, *p* < 0.01. “#” indicates a significant difference between the MB group and the MA group at the same incubation time (*p* < 0.05).

For the addition of *B. subtilis* (experiment III), ^*^p < 0.05 and ^**^p < 0.01 were used to indicate the effects of different concentrations and different states of *B. subtilis* on the expression levels of each key factor in the PIK3CB–AKT–mTORC1 signaling pathway in rumen explants (compared to the control). ^#^*p* < 0.05 was used to indicate the effects of living or inactivated bacteria on gene expression in the pathway.

For the addition of LY294002 and rapamycin, ^*^p < 0.05 and ^**^p < 0.01 were used to indicate the effects of different inhibitors on the expression levels of each key factor in the PIK3CB–AKT–mTORC1 signaling pathway in rumen explants (compared to the control).

## Results

### Separation, Identification, and Biological Characteristics of *B. subtilis*

**Figure 1:**
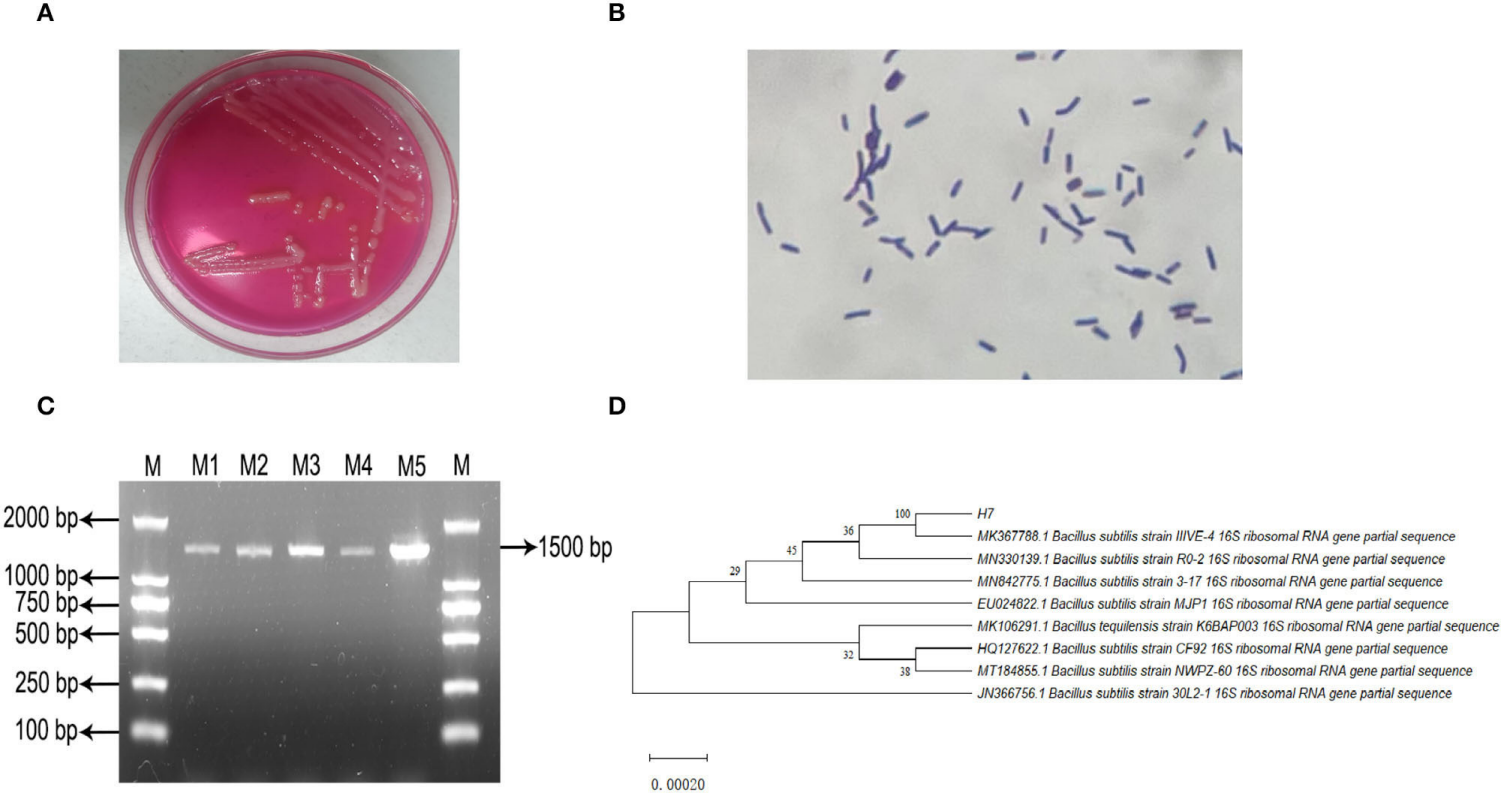
Isolation, incubation and identification of *Bacillus subtilis* IIIVE-4. **(A)** Mannitol polymyxin plate colony morphology. **(B)** Gram staining test results. **(C)** PCR amplification product electrophoresis; M: DL2 000 Marker; M1 to M5: IIIVE-4 DNA amplification products. **(D)** phylogenetic tree; H7 is the *Bacillus subtilis* IIIVE-4 gene sequence; values of developmental tree nodes represent Bookstrap values; numbered values are the GenBank database accession numbers.

Microscopic observations revealed that the isolated strain *B. subtilis* IIVE-4 was a gram-positive rod-shaped bacterium with an overall oval or rod-like morphology. Soft, slightly yellowish colonies were formed after incubation on Mannitol-Egg-Yolk-Polymyxin Agar Base at 37°C for 24 h (Figures 1A,B). An amplified gene fragment of 1,500 bp was obtained through bacterial 16S rDNA electrophoresis, and sequence analysis *via* GenBank indicated that the isolated bacteria were isogenous with *B. subtilis* IIIVE-4 (Figures 1C,D). *Bacillus subtilis* IIIVE-4 grew the fastest at 38°C, with growth declining at higher temperatures (Supplementary Figure 1A). After culturing in 0.5, 1.0, and 1.5% bile salts for 24 h, the survival rate *of B. subtilis* IIIVE-4 was more than 50% of the standard culture state (Supplementary Figure 1B). This suggests that *B. subtilis* IIIVE-4 could survive and grow in conditions similar to those of the small intestine-like intestinal bile concentration in healthy animals to 0.3% (31), pH range of the gastrointestinal tract in healthy animals (6.15 < pH <6.80) (32). The survival rate of *B. subtilis* IIIVE-4 increased rapidly when the culture pH was increased from 3.0 to 6.0 (Supplementary Figure 1C). When the pH values were 6.0 and 7.0, the bacterial survival rate leveled-off.


$$\begin{array}{l} Survival\;rate=(OD\;of\;bacterial\;solution\;at\;each\;time\;point\;\\ \;-OD\;of\;pure\;medium)/(maximum\;OD\;of\;bacterial\;solution\;\\ \;-OD\;of\;pure\;medium). \end{array}$$

*Bacillus subtilis* IIIVE-4 had some tolerance to pH between 3.0 and 4.0, suggesting that it could survive in the animal gastrointestinal tract. Similarly, when the pH of the medium was >7.0, *B. subtilis* IIVE-4 tended to first increase and then decrease to a stable point, indicating resistance to a strong alkali environment. The survival rate was highest at pH 7.5.

### Incubation and Activity of Rumen Explants

**Figure 2:**
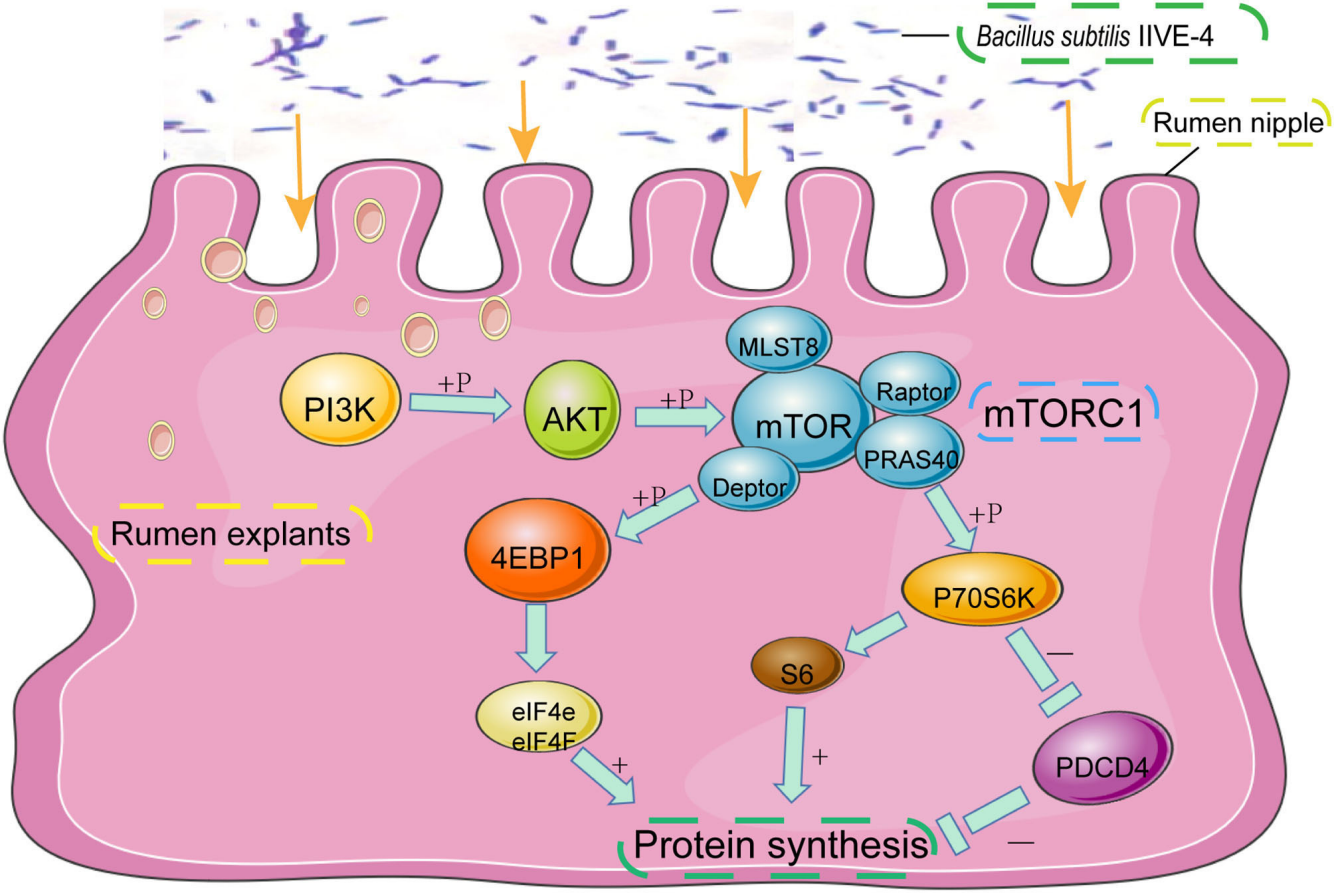
The protective mechanism of *Bacillus subtilis* can promote protein synthesis in rumen explants by the PIK3CB-AKT-mTORC1 pathway. In the rumen of Holstein cows, *Bacillus subtilis* IIIVE-4 was able to secrete nutrients (methionine, leucine, etc.) to enhance protein synthesis by acting on the PIK3CB-AKT-mTORC1 signaling pathway and increasing the expression levels of 4EBP1 and P70S6K proteins.

Thiazolyl blue tetrazolium bromide (MTT) was used in this study to evaluate the proliferation and cellular activity of ruminal explants. The MTT curve showed that the viability of rumen tissue cells was relatively stable when the incubation time ranged from 4–12 h (Figure 2). By 16 h, there was a small decrease in cell viability. After 28 h, there was a substantial decrease in the cell viability. Cell viability was maintained at a relatively low level as incubation time continued to increase.

**Figure 3:**
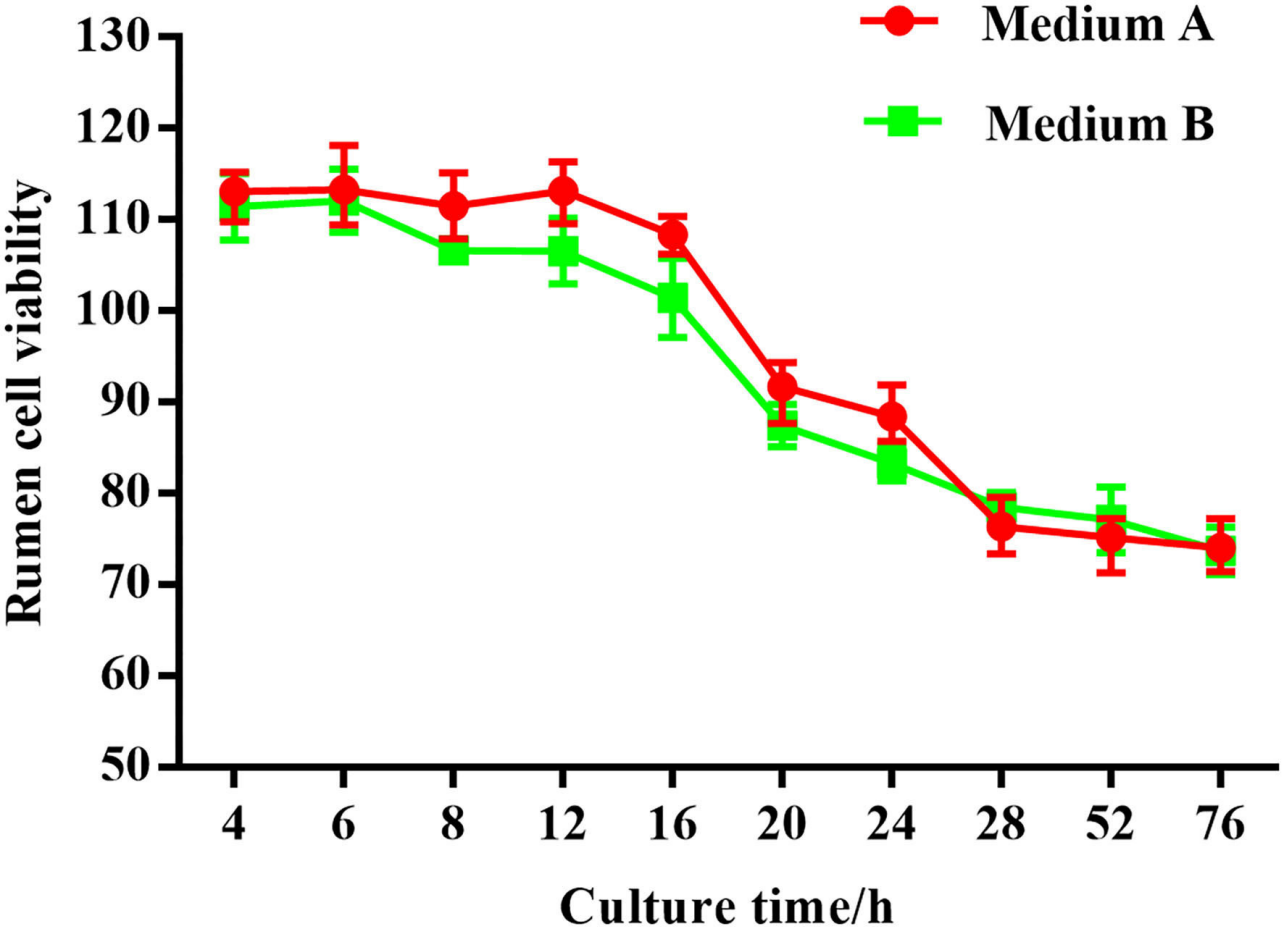
Effect of different concentrations of *Bacillus subtilis* IIIVE-4 on the cell activity of rumen explants was determined by MTT. Different concentrations of living and inactivated *Bacillus subtilis* IIIVE-4 had no significant effect on explant viability.

No structural differences were detected at any time with medium A (M.A.) or medium B (M. B.) during the first 12 h of culture (Figure 3). Starting from 16 h, regardless of M. A. or M. B. culture media, the tissue organizational structure began to disintegrate, and longer incubations led to further negative effects on tissue structure after 72 h. Thus, a 12 h incubation was deemed suitable for subsequent experiments.

**Figure 4:**
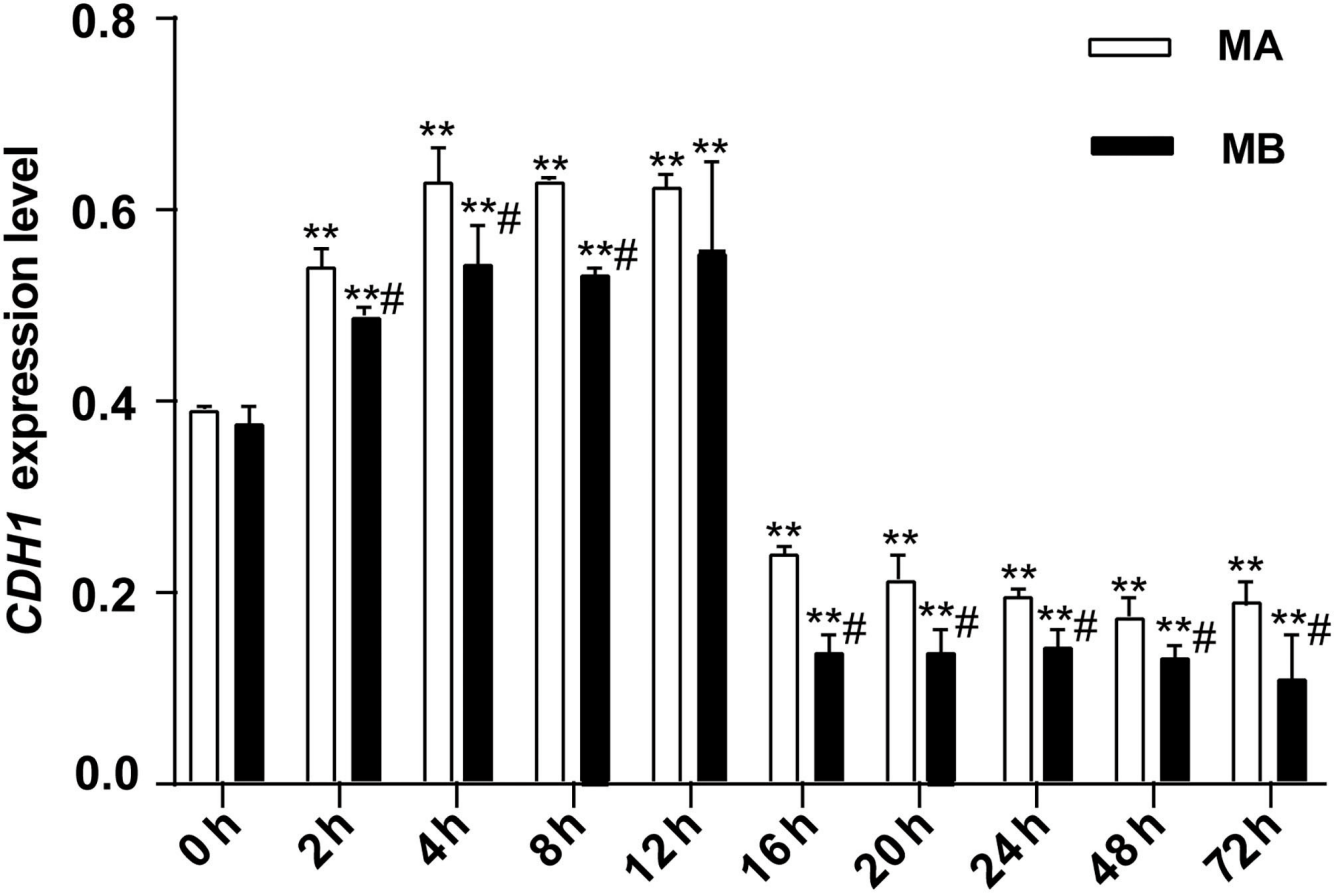
The relative expression of *CDH1* mRNA at different culture times (*n* = 9). *CDH1* expression in rumen explants was measured to determine the activity of rumen explants in different culture media and different culture times and to further determine the optimal culture time and medium for the explants. The expression of *CDH1* was stable rising before 12 h but decreasing after 16 h and remaining at a low expression level till the cultured end at 72 h. One-way ANOVA in SPSS 22.0 software was used to analyze the data, presented as mean ± sd. The ratio of all gene expression levels compared to the housekeeping gene β-actin was obtained as the relative expression of the gene. ^#^Indicates significant difference compared to other groups at the same time or the same concentration gradient (*p* < 0.05). **Indicates a highly significant difference (*p* < 0.01).

As shown in Figure 4, for 0–12 h, the expression of *CDH1*, which represents the ability of epithelial cell differentiation in explant tissue, gradually increased and then leveled-off. After 12 h of culture, expression of *CDH1* began to decrease along with the apparent viability of the tissue. Both results were consistent with the histological results. The overall trend of *CDH1* mRNA abundance in tissue was the same in culture media A or B, with or without fetal bovine serum. However, at each time point, *CDH1* was more highly expressed in medium A than in medium B. Thus, culture medium A was used in subsequent experiments.

### *B. subtilis*-Stimulated Expression of RNA and Protein in Rumen Explants

**Figure 5:**
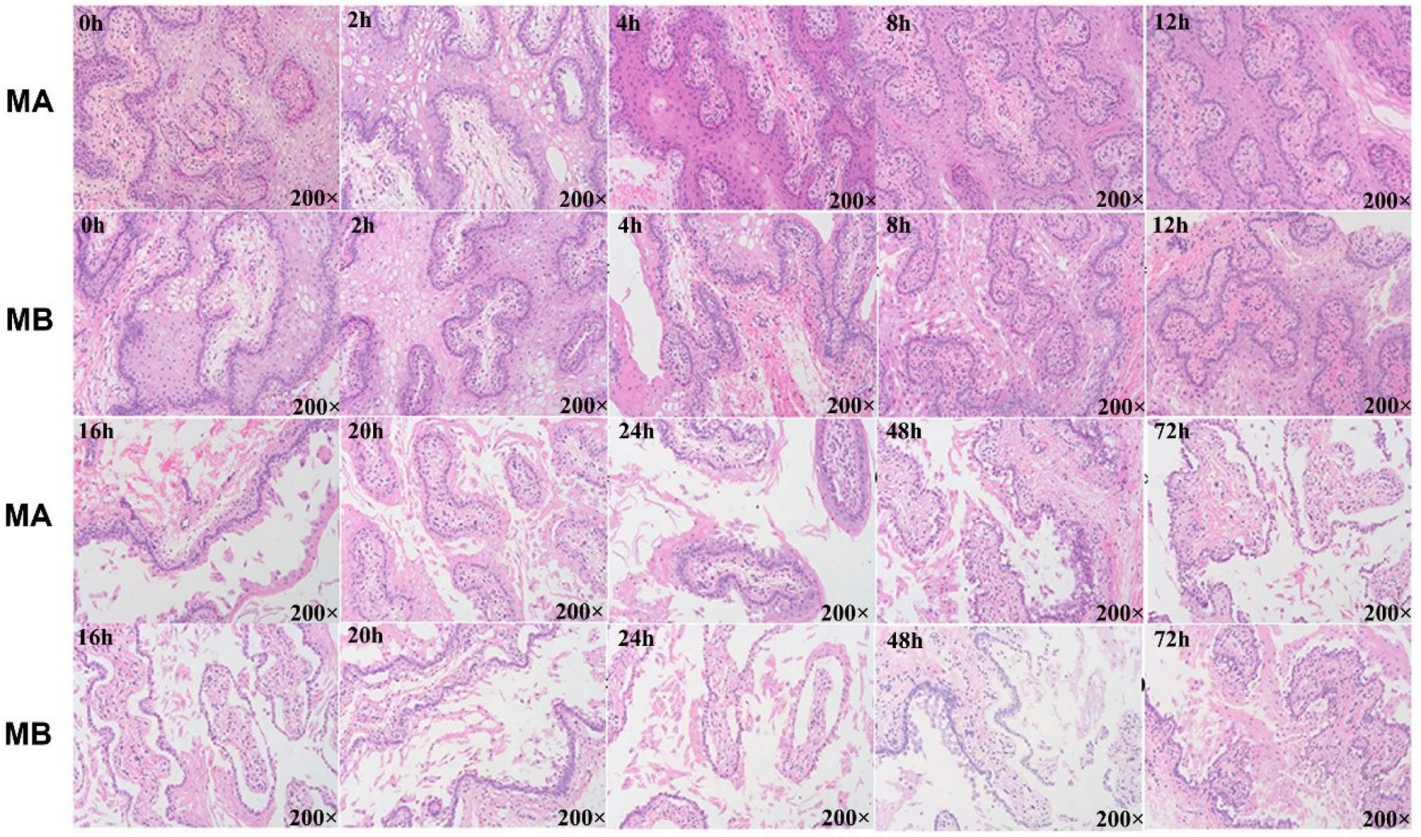
Histology results of rumen explant tissue with different media and culture time. Hematoxylin–eosin staining (HE) or HE staining is a common method for observing the morphology and structural integrity of tissue cells. Normal physiological changes of animal explants in general or morphological changes after the addition of certain stimuli can be observed by the HE staining method. MA is rumen explant cultured in medium A containing 10% serum, and MB is rumen explant cultured in medium B without serum. The effect of different media and cultural times on the growth of rumen explants was observed by the integrity of the flat cut structure of the rumen epithelial tissue. The explant maintained a complete organizational structure at 12 h but started to disintegrate after 16 h and festering increased with longer cultural time.

**Figure 6:**
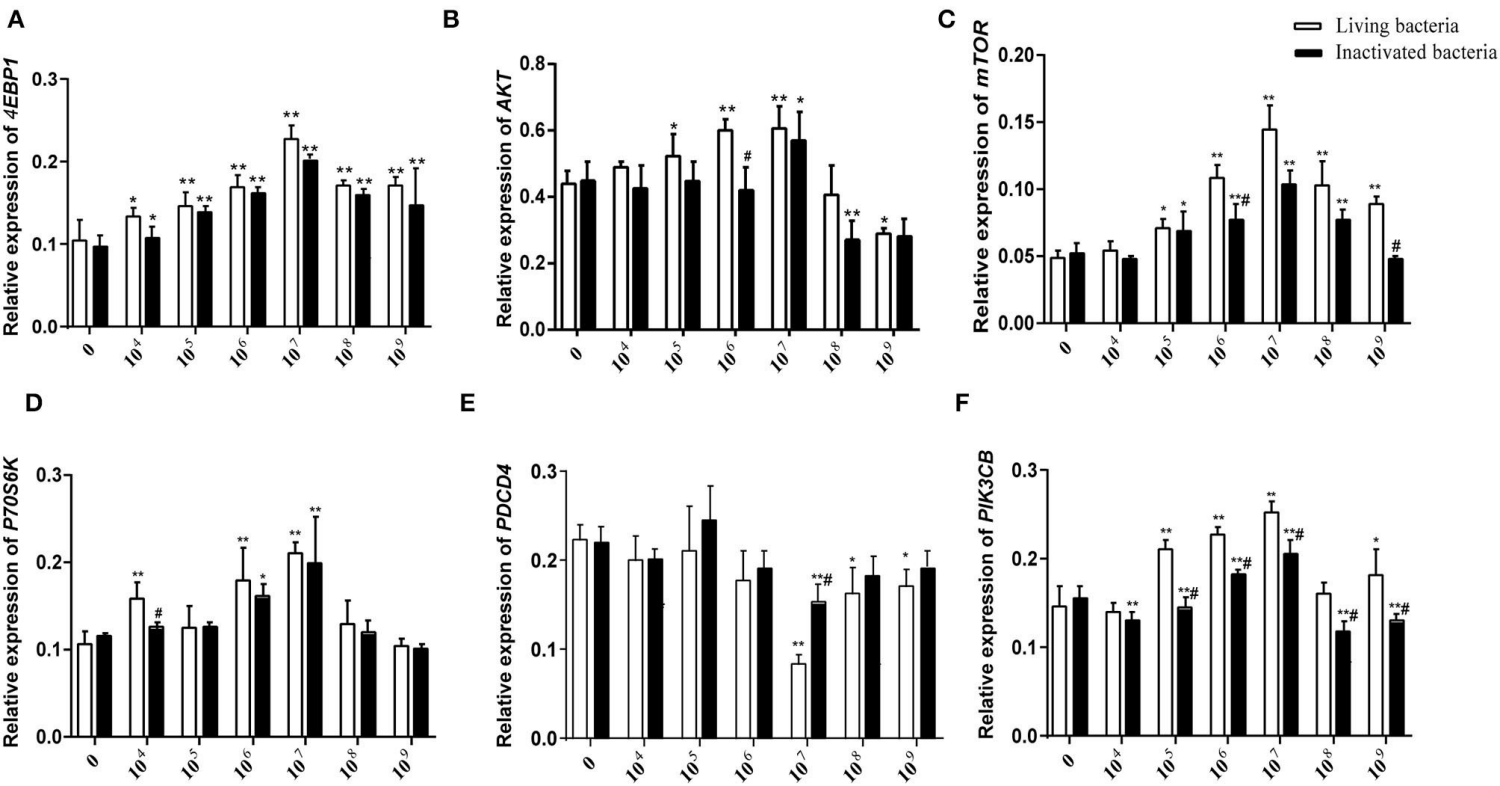
Effects of different concentrations of living IIIVE-4 bacteria on gene expression of key factors in the PIK3CB-AKT-mTORC1 pathway in rumen explants (*n* = 9). The expression of *4EBP1* **(A)**, *AKT* **(B)**, *mTOR4* **(C)**, *P70S6K* **(D)**, and *PI3K* **(F)** genes was highest when adding living IIIVE-4 bacteria at a concentration of 10^7^, while *PDCD* **(E)** gene expression was lowest when the bacterial concentration was 10^7^. One-way ANOVA in SPSS 22.0 software was used to analyze the data, presented as mean ± sd. The ratio of all gene expression levels compared to the housekeeping gene β-actin was obtained as the relative expression of the gene. *Indicates significant difference from control group within the same group (*p* < 0.05). ^#^Indicates significant difference compared to other groups at the same time or the same concentration gradient (*p* < 0.05). **Indicates highly significant difference (*P* < 0.01).

**Figure 7:**
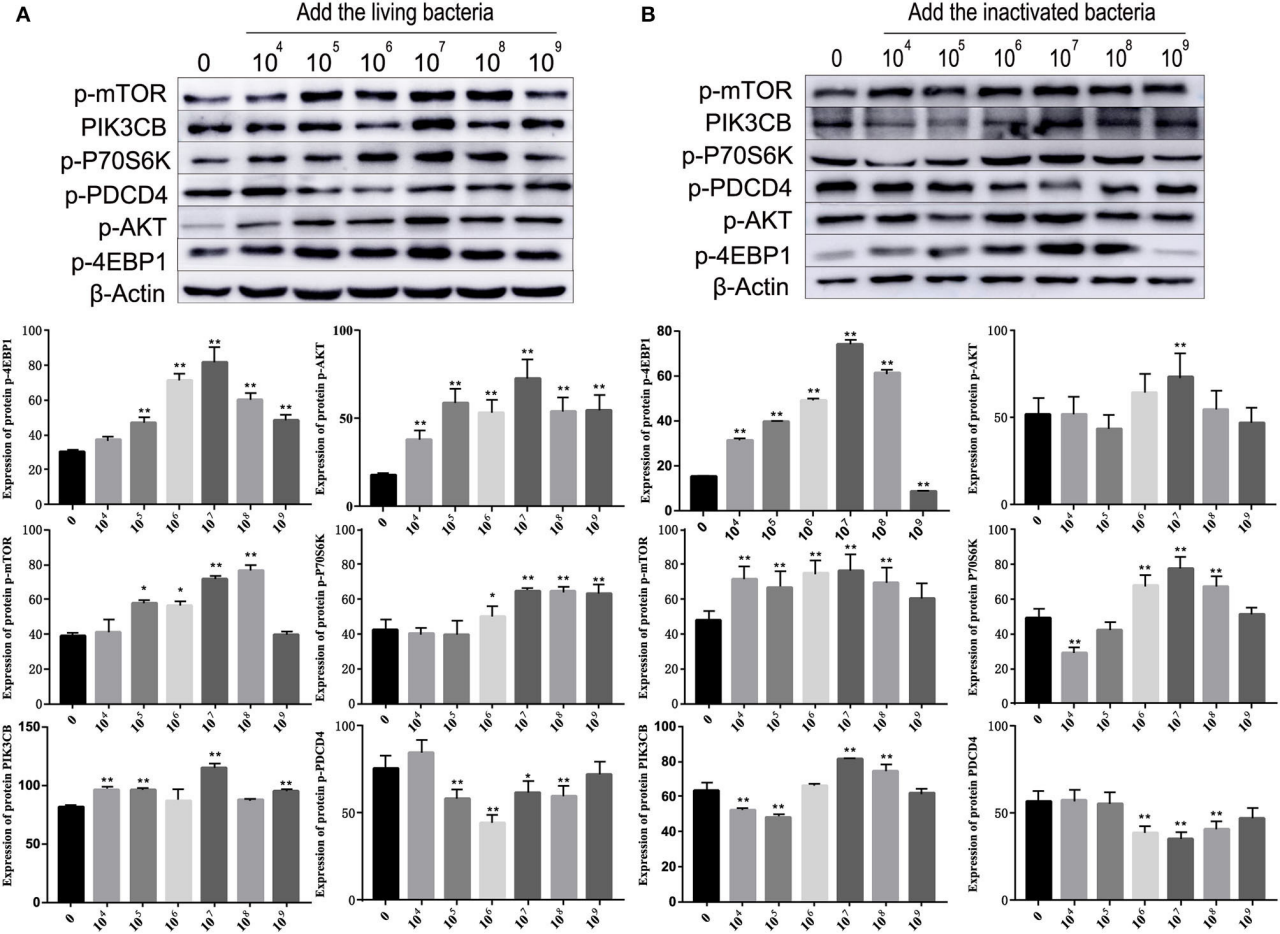
Effects of different concentrations of living bacteria *Bacillus subtilis* IIIVE-4 **(A)** and inactivated bacteria *Bacillus subtilis* IIIVE-4 **(B)** on protein expressions in the PIK3CB-AKT-mTORC1 pathway in rumen explants (*n* = 9). The protective mechanism of 2.0 × 10^7^ cfu/ml *B. subtilis* can promote protein synthesis in rumen explants by the PIK3CB-AKT-mTORC1 pathway. In the rumen of Holstein cows, 2.0 × 10^7^ cfu/ml *B. subtilis* IIIVE-4 was able to secrete nutrients (methionine, leucine, etc.) to enhance protein synthesis by acting on the PIK3CB-AKT-mTORC1 signaling pathway and increasing the expression levels of 4EBP1 and P70S6K proteins. The expression of PIK3CB, p70s6k, AKT, and 4EBP1 proteins was highest when adding live IIIVE-4 bacteria at a concentration gradient of 10^7^ cfu/ml, while mTOR proteins reached their highest expression at 10^8^ cfu/ml and PDCD4 at 10^6^ cfu/ml, but P70S6k and 4EBP1, which are more closely related to protein synthesis, reached their highest at a concentration gradient of 2.0 × 10^7^ cfu/ml, so 2.0 × 10^7^ cfu/ml was determined as the optimal bacterial solution concentration. One-way ANOVA in SPSS 22.0 software was used to analyze the data, presented as mean ± sd. The ratio of all gene expression levels compared to the housekeeping gene β-actin was obtained as the relative expression of the gene. *Indicates significant difference from control group within the same group (*p* < 0.05). **Indicates a highly significant difference (*p* < 0.01).

As shown in Figure 5, different concentrations of living and inactivated bacteria had no significant effect on explant viability (MTT assay). As shown in Figure 6, the addition of living bacteria and inactivated bacteria had varying degrees of influence on the expression levels of *PIK3CB, AKT, mTOR, P70S6K1*, and *4EBP1* when the bacterial concentration was 2.0 × 10^7^ CFU/ml, while the expression of *PDCD4* was lowest. Moreover, the expression levels of *PIK3CB, AKT, mTOR*, and *P70S6K1* genes were more pronounced with the addition of living bacteria than with the addition of inactivated bacteria. As shown in Figure 7, the expression of target proteins at different concentrations of living (Figure 7A) and inactivated bacteria (Figure 7B) was consistent with gene expression. For instance, PIK3CB, p-AKT, p-mTOR, p-P70S6K1, and p-4EBP1 protein expression was the highest at a bacterial concentration of 10^7^ CFU/ml, and PDCD4 was the lowest, suggesting an opposite relationship between protein synthesis and apoptosis. The highest protein and gene expression were observed at a bacterial concentration of 10^7^ CFU/ml.

**Figure 8:**
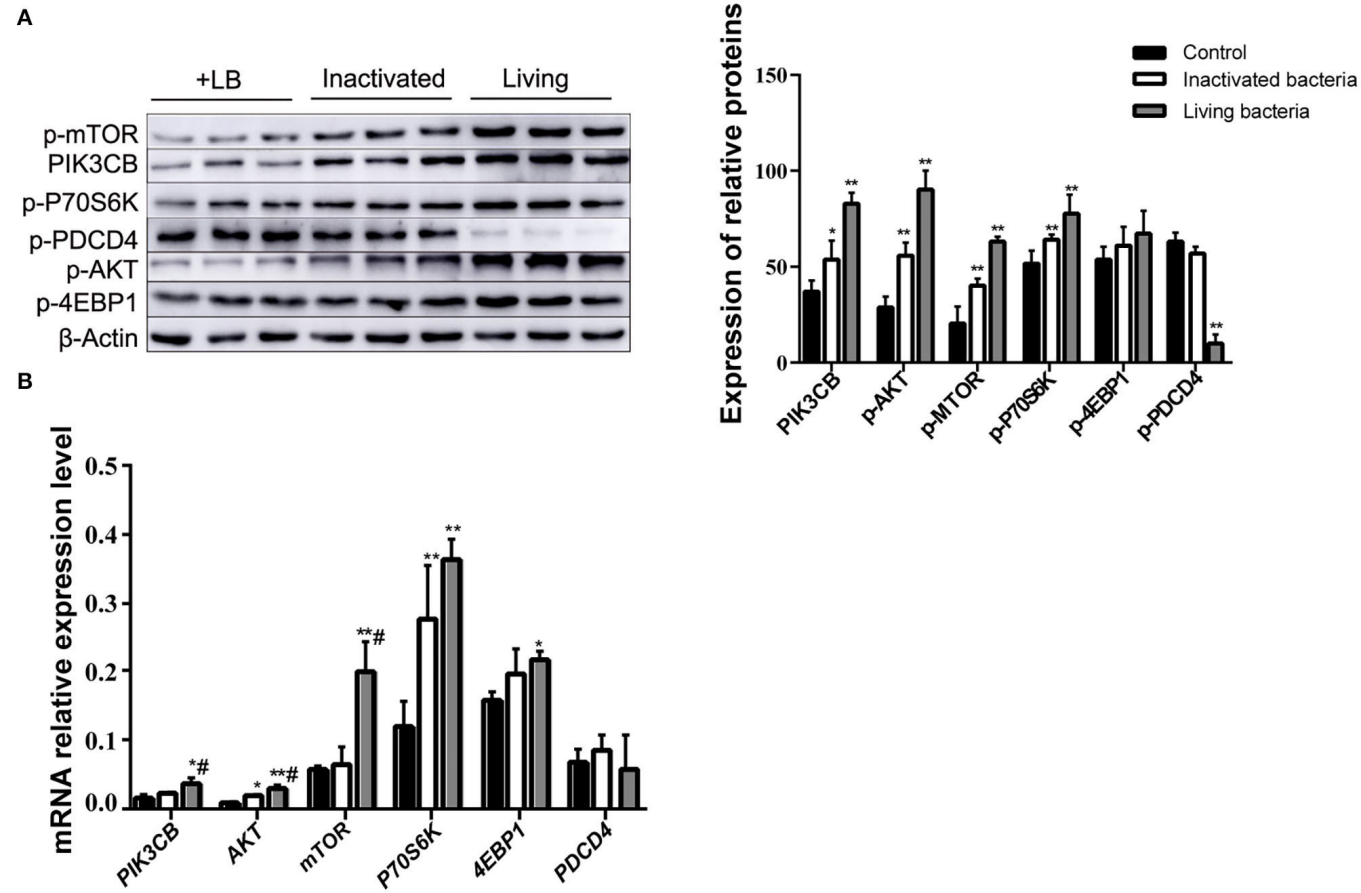
Expression of proteins **(A)** and genes **(B)** in the rumen explants with living and inactivated bacteria (*n* = 9). One-way ANOVA in SPSS 22.0 software was used to analyse the data, presented as mean ± sd. The ratio of all gene expression levels compared to the housekeeping gene β-actin was obtained as the relative expression of the gene. *Indicates significant difference from control group within the same group (*p* < 0.05). ^#^Indicates significant difference compared to other groups at the same time or the same concentration gradient (*p* < 0.05). **Indicates a highly significant difference (*p* < 0.01).

To further clarify the differences in the effects of *B. subtilis* and inactivated bacteria on protein synthesis, we compared the results of the effects of living and inactivated bacteria at a concentration of 2.0 × 10^7^ CFU/ml on key proteins in the PI3K–AKT–mTORC1 signaling pathway, and the results are shown in Figure 8. The results from Figure 8 showed that both living and heat-inactivated *B. subtilis* at 2.0 × 10^7^ CFU/ml were able to exert a more pronounced effect on key factors in the pathway, and there was a significant difference in the effect on the expression levels of key proteins between living and heat-inactivated bacteria (except for 4EBP1), while there was a significant difference in the expression levels of *PI3K, AKT*, and *mTOR* genes. The effects of key proteins and genes in the pathway were greater in the living bacteria group than in the inactivated bacteria group (*p* < 0.05). The results indicated that protein and gene expression in response to inactivated bacteria was no less than that of living bacteria. The effect of adding living bacteria on proteins and genes was significantly higher than that of the control.

**Figure 9:**
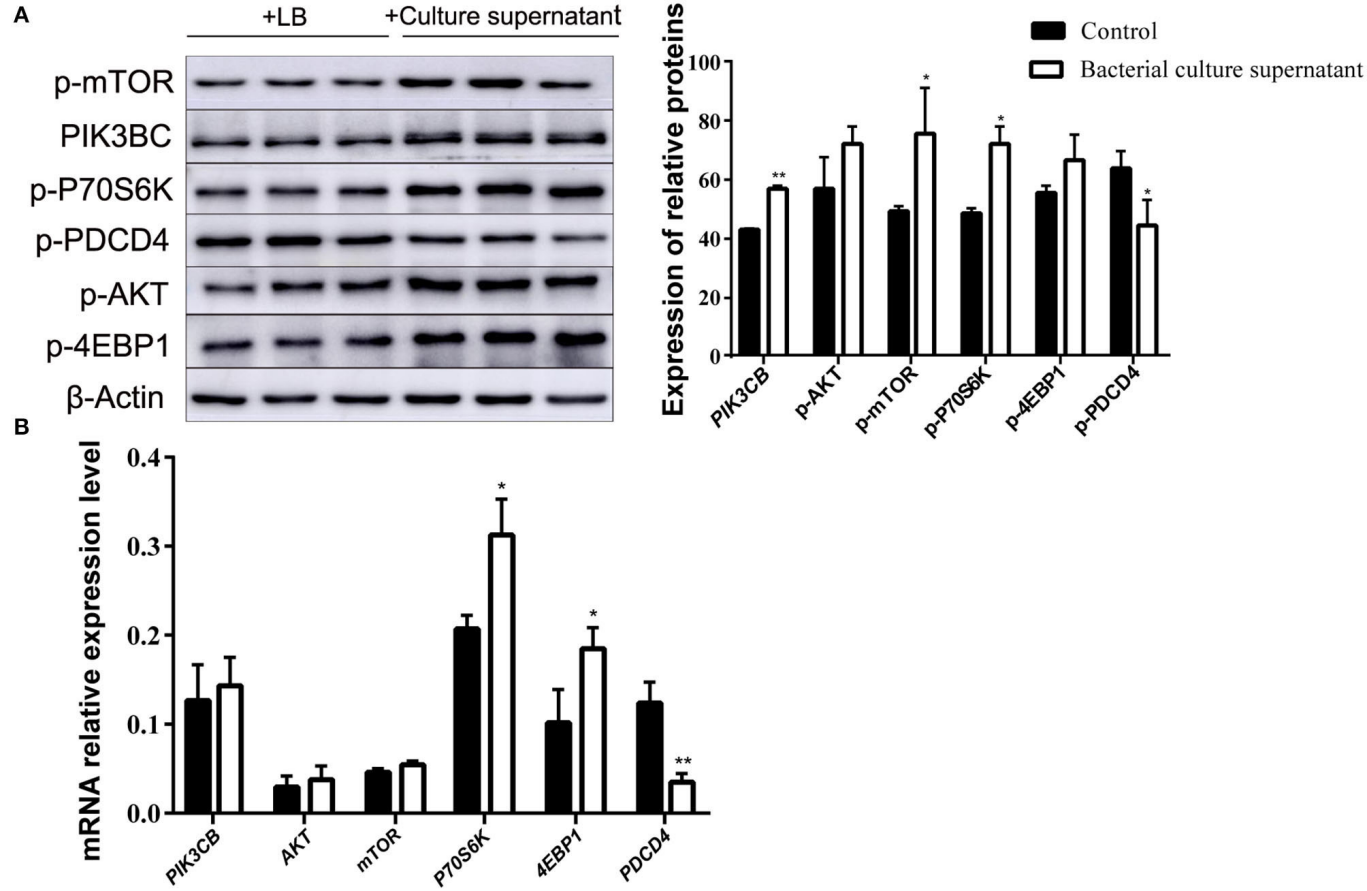
Expression of proteins **(A)** and genes **(B)** in the rumen explants with supernatant of inactivated bacteria (*n* = 9). One-way ANOVA in SPSS 22.0 software was used to analyse the data, presented as mean ± sd. The ratio of all gene expression levels compared to the housekeeping gene β-actin was obtained as the relative expression of the gene. *Indicates significant difference from control group within the same group (*p* < 0.05). **Indicates highly significant difference (*P* < 0.01).

The addition of supernatant from cultures receiving inactivated bacteria upregulated the expression of *PIK3CB* and *AKT* (Figure 9A). Only *P70S6K* and *4EBP1* were significantly upregulated, whereas *PDCD4* was downregulated. With the exception of PDCD4, the expression of all other proteins evaluated was upregulated by the addition of supernatant from inactivated bacteria during the 12 h incubations (Figure 9B).

**Figure 10:**
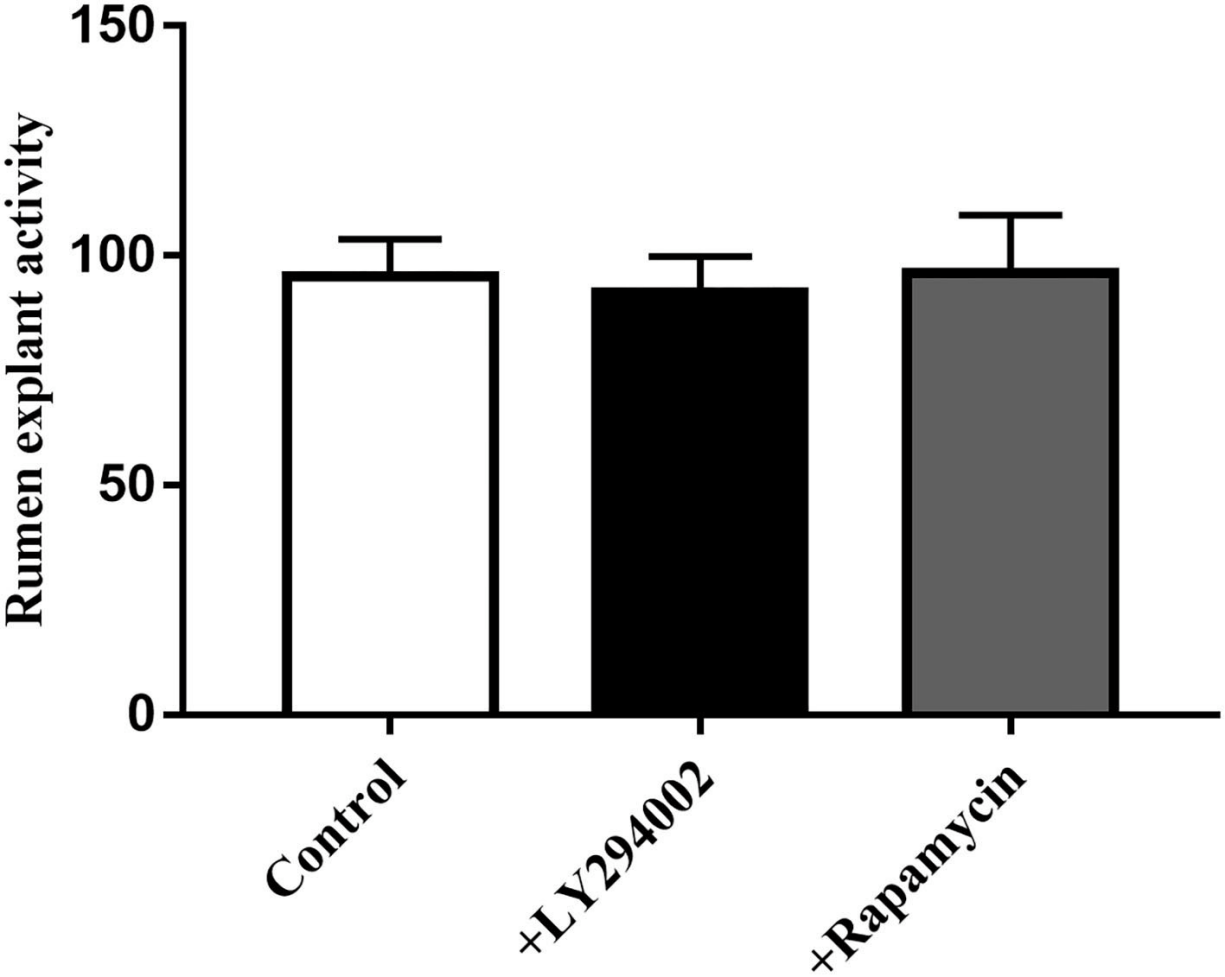
Effect of LY294002 and Rapamycin on the cell activity of rumen explants was determined by MTT. The MTT data indicated that the addition of LY294002 or Rapamycin did not affect viability or had toxic effects on rumen explants. One-way ANOVA in SPSS 22.0 software was used to analyze the data, presented as mean ± sd.

**Figure 11:**
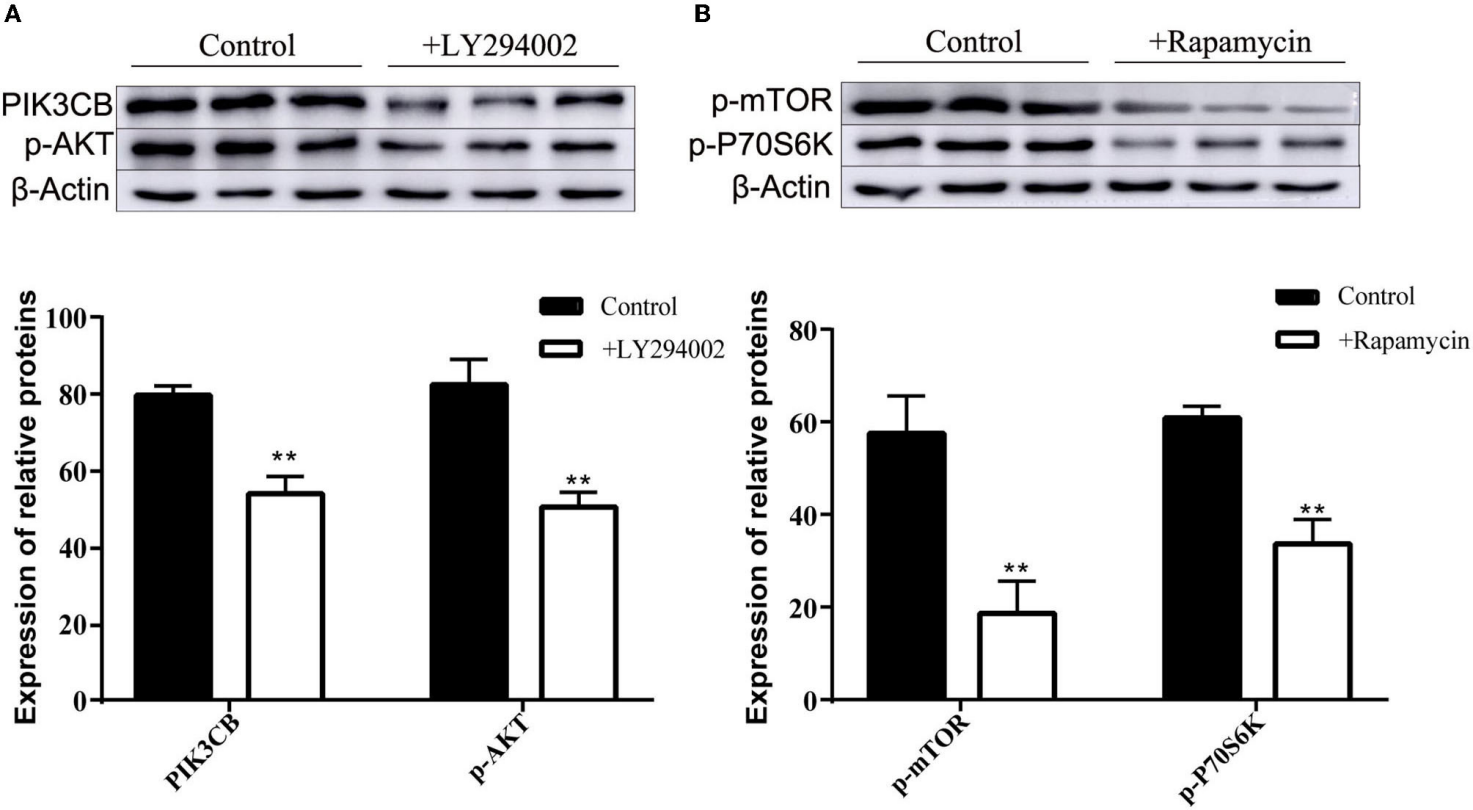
Protein expression with adding inhibitor LY294002 **(A)** and Rapamycin **(B)** in tissue culture (*n* = 9). One-way ANOVA in SPSS 22.0 software was used to analyze the data, presented as mean ± sd. The ratio of all gene expression levels compared to the housekeeping gene β-actin was obtained as the relative expression of the gene. **Indicates highly significant difference (*p* < 0.01).

MTT data indicated that the addition of LY294002 or rapamycin did not affect viability or had toxic effects on rumen explants (Figure 10). A significant decrease in protein expression was detected after the addition of LY294002 and rapamycin. Similarly, the expression of PIK3CB, mTOR, AKT, and P70S6K was markedly inhibited (Figure 11).

### Analysis of Free Amino Acid Content in the Supernatant of Explants Culture

As shown in Table 1, glutamic acid, glycine, phenylalanine, histidine, arginine, and dl-proline had the highest content in the control group and were significantly different from the other two groups. The highest levels of tyrosine and cysteine were detected in the supernatants of the living bacterial culture group. The aspartic acid, threonine, serine, d-alanine, valine, methionine, isoleucine, n-carbobenzoxy-dl-leucine, and lysine contents in the culture supernatant of inactivated bacteria were the highest and differed significantly from the other two groups.

**Table 1:** Determination of 17 kinds of free amino acids in the supernatant of bacterial culture (*n* = 9, mg/mL).

**Item**	**Control**	**Living supernatant**	**Inactivated supernatant**
	**Mean ±SD**	**Mean ±SD**	**Mean ±SD**
Aspartic acid	0.0442 ± 0.0042^c^	0.0887 ± 0.0006^b^	0.1387 ± 0.0012^a^
Threonine	0.0377 ± 0.0009^c^	0.0887 ± 0.0007^b^	0.1415 ± 0.0005^a^
Serine	0.2343 ± 0.0005^b^	0.2323 ± 0.0012^b^	0.2760 ± 0.0045^a^
Glutamic acid	0.4433 ± 0.0018^a^	0.2409 ± 0.0009^b^	0.1512 ± 0.0009^c^
Glycine	0.0558 ± 0.0005^a^	0.0528 ± 0.0006^b^	0.0333 ± 0.0018^c^
D-Alanine	0.1580 ± 0.0007^c^	0.2524 ± 0.0013^b^	0.4018 ± 0.0027^a^
Valine	0.1369 ± 0.0008^c^	0.2198 ± 0.0006^b^	0.2241 ± 0.0052^a^
Methionine	0.0680 ± 0.0007^c^	0.1089 ± 0.0011^b^	0.1269 ± 0.0018^a^
Isoleucine	0.1116 ± 0.0006^c^	0.1792 ± 0.0010^b^	0.1913 ± 0.0009^a^
N-carbobenzoxy-dl-leucine	0.3008 ± 0.0061^c^	0.4858 ± 0.0020^b^	0.6040 ± 0.0022^a^
Tyrosine	0.0787 ± 0.0008^c^	0.0205 ± 0.0006^a^	0.0158 ± 0.0002^b^
Phenylalanine	0.3769 ± 0.0007^a^	0.2566 ± 0.0006^b^	0.1597 ± 0.0011^c^
Histidine	0.2007 ± 0.0009^a^	0.1096 ± 0.0008^b^	0.0693 ± 0.0008^c^
Lysine	0.0109 ± 0.0003^b^	0.0095 ± 0.0006^b^	0.5552 ± 0.0029^a^
Arginine	0.3553 ± 0.0013^a^	0.0378 ± 0.0009^b^	0.0238 ± 0.0005^c^
Dl-Proline	0.0221 ± 0.0008^a^	0.0147 ± 0.0028^b^	0.0105 ± 0.0006^c^
Cysteine	0.1219 ± 0.0016^c^	0.1573 ± 0.0011^a^	0.1333 ± 0.0007^b^

*Different superscript letters within the same line indicate significant differences (p < 0.05)*.

**Figure 12:**
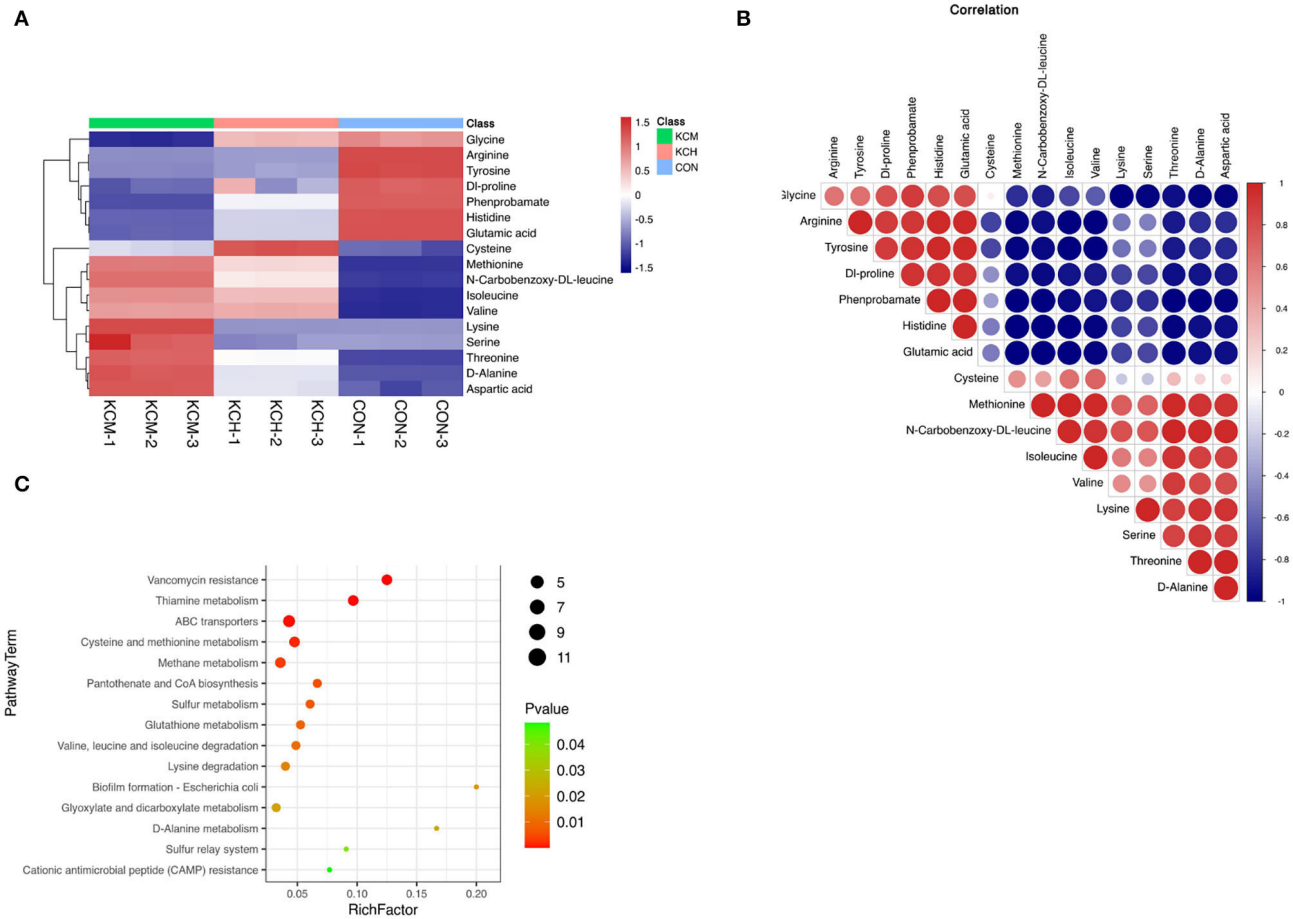
Analysis of different amino acid metabolites in the bacterial culture supernatant. The hierarchical clustering of the top 50 differential metabolites expression in the bacterial culture supernatant **(A)**, the correlation analysis of differential metabolites **(B)**, and the enrichment analysis of differential metabolite pathways **(C)**.

Hierarchical clustering of the top 17 amino acids differentially affected metabolites in the bacterial culture supernatant (Figure 12A) indicated that the contents of glycine, methionine, n-carbobenzoxy-dl-leucine, isoleucine, valine, lysine, serine, threonine, alanine, and aspartic acid in the supernatant of inactivated bacteria were greater than those in the living bacteria and LB control group.

The lysine and serine contents in the living bacterial supernatant and LB control group were similar. In contrast, the content of cysteine in the supernatant of living bacteria was the highest among the three groups, followed by that in the supernatant of inactivated bacteria, and was lowest in the control group. Among the 17 free amino acids, the content of glycine, arginine, tyrosine, dl-proline, phenylalanine, histidine, and glutamic acid in the control group was the highest, with the content of arginine and tyrosine in the supernatant of living and inactivated bacteria being similar. The contents of glycine, dl-proline, phenylalanine, histidine, and glutamic acid were the lowest in the supernatant of inactivated bacteria.

Correlation analysis (Figure 12B) showed that methionine was positively correlated with n-carbobenzoxy-dl-leucine, isoleucine, valine, lysine, serine, threonine, d-alanine, and aspartic acid, and had the strongest positive correlation with n-carbobenzoxy-dl-leucine and the strongest negative correlation with histidine. n-Carbobenzoxy-dl-leucine was positively correlated with isoleucine, valine, lysine, serine, threonine, d-alanine, and aspartic acid, and had the strongest negative correlation with phenprobamate. Isoleucine was positively correlated with valine, lysine, serine, threonine, D-alanine, and aspartic acid, and had the strongest positive correlation with valine and the strongest negative correlation with tyrosine.

Enrichment analysis of KEGG pathways (Figure 12C) showed that aminoacyl tRNA biosynthesis, cyanoamino acid metabolism, monobactam biosynthesis, glycine, serine, and threonine metabolism, valine, n-carbobenzoxy-dl-leucine, and isoleucine biosynthesis were among the most represented within the list of affected metabolites. Among the free amino acids that were significantly affected, the aminoacyl tRNA biosynthesis pathway had the highest coverage. Cyanoamino acid metabolism; monobactam biosynthesis; and glycine, serine, and threonine metabolism were among the top pathways enriched with amino acids. Valine, n-carbobenzoxy-dl-leucine, and isoleucine biosynthesis were among the most represented within the list of affected metabolites.

## Discussion

*Bacillus subtilis* IIIVE-4, isolated from dairy cow rumen fluid in the present study, demonstrated tolerance to acids and bile salts, suggesting that it might be suitable as a feed additive in fields (33). The present data, thus, agreed with those of Yun et al. (34) and indicated that tolerance of the strain to bile salts could be attributed to the action of bile salt hydrolase, an enzyme produced by probiotic bacteria (35). These data suggest that bile salt hydrolase enhances the ability of strains to colonize the intestinal tract (36). Thus, the strong tolerance of *B. subtilis* IIIVE-4 to bile salts and acids, along with the lack of toxic effects against rumen tissue, underscores the potentially beneficial symbiosis when fed to ruminants (37).

Ruminal explants in the present study, as demonstrated with sheep (20), were cultured successfully and retained the typical mucosal architecture, as evidenced by histology and *CDH1* gene expression. *CDH1*. Thus, this model was deemed suitable to address the objectives of the present study. Furthermore, the results of the rumen explant activity assay showed that the optimal incubation time for rumen explants in dairy cows was 12 h, which is lower than the 24 h reported in other studies (38). This may be the result of differences between sheep and cow species, as sheep rumen epithelial cells can continuously and stably pass through generations. A continuous increase in rumen explant activity was observed between 0 and 4 h of incubation. This was because of a decrease in explant viability due to the absence of nutrient supply during the time between isolation from the rumen epithelial tissue and placement into the medium. When placed in the medium, rumen explants rapidly absorbed nutrients and began to gradually recover viability, similar to the phenomenon observed in the study by Girard et al. (39). The establishment of a rumen explant model is important for studying the nutrient metabolism function of bacteria and the ruminant rumen. Zhang et al. used sheep rumen tissue to establish a rumen explant model and found that the optimal culture time for explants was 24 h. It is believed that a medium without fetal bovine serum is more favorable for the culture of sheep rumen epithelial tissue (20). In the present study, the addition of fetal bovine serum was found to be more favorable for the increase in explant activity, which was confirmed by Alan et al. who concluded that the addition of fetal bovine serum was beneficial in delaying the loss of explant activity (40). Furthermore, the results of the rumen explant activity assay showed that the optimal incubation time for rumen explants in dairy cows was 12 h, which is lower than the 24 h reported in other studies (38). This may be the result of differences between sheep and cow species, as sheep rumen epithelial cells can continuously and stably pass through generations. A continuous increase in rumen explant activity was observed between 0 and 4 h of incubation. This was because of a decrease in explant viability due to the absence of nutrient supply during the time between isolation from the rumen epithelial tissue and placement into the medium. When placed in the medium, rumen explants rapidly absorbed nutrients and began to gradually recover viability, similar to the phenomenon observed in the study by Giard et al. (39).

As research on probiotics continues to intensify, cellular components or metabolites of probiotics have been reported to exert an immune response, enhance disease protection, and stimulate the expression of related genes (41). For example, intracellular products of *B. subtilis* and *Lactobacillus plantarum* have potential as immunostimulants (42). The degradation of ochratoxin A by heat-inactivated *B. subtilis* was also found to be higher than that by living *B. subtilis* (43). Moreover, both living and heat-inactivated *B. subtilis* can increase the expression of PGRP-like proteins, trigger the Toll-like receptor (TOLL) pathway, and increase reactive oxygen species (ROS) production (44). Heat-inactivated *B. amyloliquefaciens* can reduce aphid resistance and can be used as a new pesticide for aphid control (45). Moreover, the culture supernatant of *B. polymyxa* has a strong inhibitory effect on pathogenic microorganisms, such as *Aeromonas* and *Pseudomonas* (46). The data in this study demonstrated that *B. subtilis* IIIVE-4 can elicit strong positive effects on rumen tissue molecular responses associated with protein synthesis. Both mRNA and protein expression of specific targets responded in the same fashion to living and inactivated bacteria, suggesting that this organism or its metabolites can promote protein synthesis in part through the PIK3CB–AKT–mTORC1 pathway (47). In this context, it should be highlighted that living and inactivated bacteria have similar potent effects on promoting protein synthesis in the rumen tissue.

Since the expression of key factors in the PI3K-AKT-mTORC1 pathway can be influenced by living and inactivated IIIVE-4 bacteria as well as the supernatant of inactivated bacteria, it is suggested that some metabolic derivatives in IIIVE-4 cultures may be involved in this process, in addition to the role of the IIIVE-4 organism itself. The results confirmed this idea, with differences in the contents of some amino acids in the supernatants of all three culture groups. Among them, Met, Ile, and Leu were the most abundant in the supernatants of inactivated cultures among the three groups. Ile and Leu can independently regulate the mTOR signaling pathway, and Ile can exert a positive effect on the phosphorylation of mTOR (48). It indicates that Ile and Leu may be one of the factors affecting the expression of key factors in the pathway. The experimental results also indicate that the addition of inactivated bacterial culture supernatant can affect the expression of proteins and genes in the PI3K-AKT-mTORC1 pathway, which is consistent with Anthony's conclusion that Met, Ile, and Leu are all able to promote protein synthesis by affecting the mTORC1 complex (49). Castro's study also confirmed this idea, suggesting that mTOR is more sensitive to Met, Ile, Thr, and Leu and can transduce synthetic signals to larger protein synthesis processes (18).

**Figure 13:**
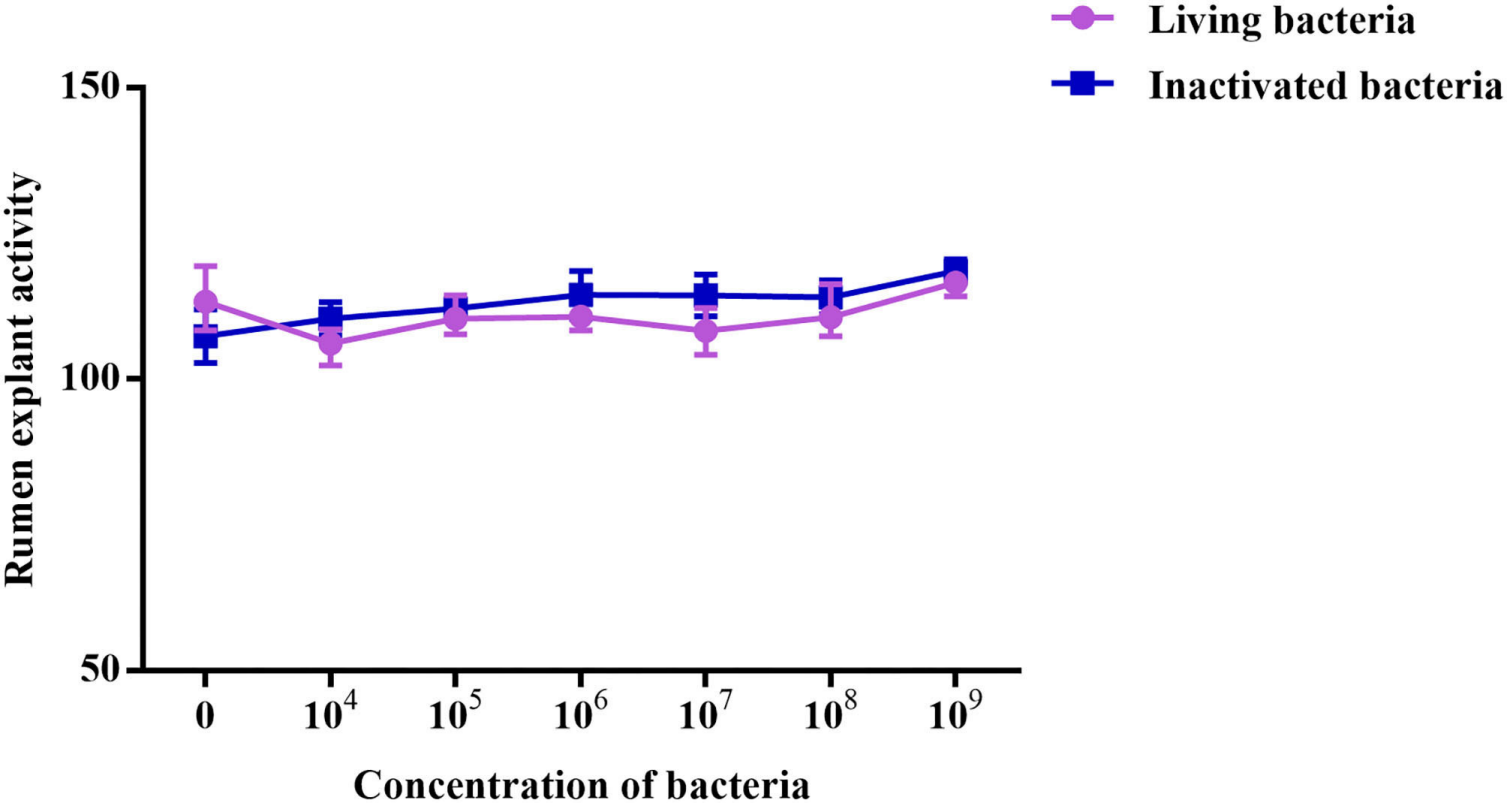
Rumen explants cell activity detected by MTT.MA is rumen explant cultured in medium A containing 10% serum, MB is rumen explant cultured in medium B without serum. The data are presented as mean ± sd. The MTT curve shows that the viability of rumen tissue cells maintained a relatively stable level when the incubation time was between 4 and 12 h. By 16 h, there was a small decrease in cell viability. At 28 h, there was a substantial decrease in cell viability. The cell viability was maintained at a relatively low level as the incubation time continued to increase.

The content of the remaining 14 free amino acids also differed among the three groups, and these differential amino acids may be one of the factors affecting protein synthesis. For example, Thr promotes mTOR phosphorylation (50). Burgos showed that when all amino acids were removed from the medium, the phosphorylation levels of many substrates of mTORC1 were significantly reduced, slowing down the protein synthesis process (19). However, the specific pathways of action of these amino acids in cows remain unclear. In this study, only 17 free amino acids were found to be mainly enriched in pathways, such as aminyl-tRNA synthesis and cyanogenic amino acid metabolism, whereas there were different degrees of correlation among Leu, Ile, Val, Lys, and Ser amino acids and deeper pathways of action are yet to be investigated. As shown in Figure 12, methionine, isoleucine, and leucine in the supernatant from the inactivated bacterial culture were the highest among the three groups, and they may directly regulate the mTOR signaling pathway in rumen tissues (19, 48). For example, n-carbobenzoxy-dl-leucine, isoleucine, and threonine can promote the phosphorylation of mTOR (50). Thus, inactivated bacterial cultures could also affect the expression of critical factors in protein synthesis pathways in the rumen tissues. *Bacillus subtilis* IIVE-4 may also be potentially involved in other pathways that promote protein synthesis (Figure 13).

## Conclusion

We isolated *B. subtilis* and confirmed that living and inactivated *B. subtilis* can promote protein synthesis in ruminal tissue explants by altering the expression levels of related factors in the PIK3CB–AKT–mTORC1 pathway. In addition, we demonstrated that the *in vivo* ruminal tissue culture system is a suitable model for studying probiotic-induced alterations in tissue functions. This study provides insights into the synergism between rumen microorganisms and tissues, which work in coordination with other physiological mechanisms (such as endocrine factors and blood flow) to promote tissue protein synthesis, thus forming the basis for future mechanistic studies related to microbial regulation and dietary supply of proteins. Such studies could further aid in optimizing feed efficiency and increasing the use of inactivated bacteria as additives in dairy cow farming.

## Data Availability Statement

The datasets presented in this study can be found in online repositories. The names of the repository/repositories and accession number(s) can be found below: https://www.ncbi.nlm.nih.gov/bioproject/811249.

## Author Contributions

QW, YC, and CX conceived the study. YR, BG, HZ, QW, YC, QJ, and CX carried out experiments and data analysis. QW, YC, and YR interpreted the data. QW and YC wrote the manuscript. JL and ZD reviewed and modified the manuscript. All authors approved the final version.

## Funding

This work was supported by Major Science and Technology project of Heilongjiang Province (Grant No. 2021ZX12B03), the Provincial Institute Cooperation Project of the Heilongjiang Science and Technology Plan (Grant Nos. YS20B04 and YS19B01), the Development Project of Local Universities in Heilongjiang Bayi Agricultural University (Grant Nos. ZRCQC201803 and ZRCLG201904), the Postdoctoral Scientific Research Developmental Fund of Heilongjiang Province (Grant No. LBH-Q20161), the National Natural Science Foundation of China (Beijing, China; Grant Nos. 32125038 and 32072931), and Graduate innovative scientific research project of Heilongjiang Bayi Agricultural University (Grant No. YJSCX2021-Y05).

## Conflict of Interest

The authors declare that the research was conducted in the absence of any commercial or financial relationships that could be construed as a potential conflict of interest.

## Publisher's Note

All claims expressed in this article are solely those of the authors and do not necessarily represent those of their affiliated organizations, or those of the publisher, the editors and the reviewers. Any product that may be evaluated in this article, or claim that may be made by its manufacturer, is not guaranteed or endorsed by the publisher.
