# Supplementary material for: Bacillus subtilis Produces Amino Acids to Stimulate Protein Synthesis in Ruminal Tissue Explants via the Phosphatidylinositol-4,5-Bisphosphate 3-Kinase Catalytic Subunit Beta–Serine/Threonine Kinase–Mammalian Target of Rapamycin Complex 1 Pathway
Source: Front Vet Sci. 2022 Jun 27;9:852321. doi: 10.3389/fvets.2022.852321 (PMC9272757; doi:10.3389/fvets.2022.852321)
Supplement: Supplementary file 1 [file Data_Sheet_1.docx]

**Supplementary data**

**Supplemental Figure 1**


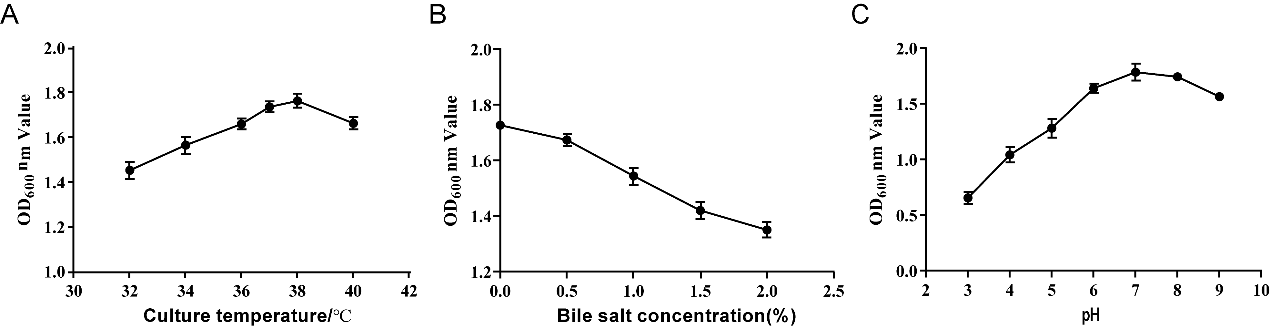


**Supplemental Figure 1.** Tolerant characters of Bacillus subtilis IIVE-4. Bacillus subtilis IIVE-4 was cultured in different culture temperature, bile salt concentration and pH to determine its tolerant characters of heat, bile, acid and alkali. Figure 1A showed Bacillus subtilis IIVE-4 growth curve at different temperature, which could grow well under 40℃, and 38℃ was the cultural temperature peak. The growth condition of the colony is different under different temperature conditions. By comparing the number of colony growth under different incubation temperature conditions, the optimal temperature for colony growth can be obtained. Figure 1B and 1C showed Bile salt and pH tolerance curve, the tolerance to bile salt and acid can reflect the survival and colonization ability of bacterial strains in the stomach and intestine. Bacillus subtilis IIVE-4 could survive with bile salt concentration up to 2.0% and it could resist pH 4, 5, and 9, best pH is 7.5.

**Supplemental Table 1.** Primers for PCR

| Gene name | Forward | Reverse |
| --- | --- | --- |
| *β-ACTIN* | GTCACCAACTGGGACGACA | AGGCGTACAGGGACAGCA |
| *mTOR* | CGAAACCCTGGATGTCCCAA | AGGACACCAGCCAATGTAGC |
| *PIK3CB* | TTAAATGGTGAGCACGGAGATG | AAATGTCACCAGACCACTCCT |
| *AKT* | TACCTTATCCCCTCAACAACT | ATTCTCTCTTCTTCTTGCCTC |
| *4EBP1* | CCCTGGAGGTACCAGGATCA | CATCGCCTGTAGGGCTAGTG |
| *P70S6K* | CGGAACAGTCACACACACCT | ACTCCACCAATCCACAGCAC |
| *PDCD4* | AAAACTCATCCCGGGACTCT | CTGCACCACCTTTCTTTGGT |
| *CDH1* | CCCCTGTCGGTGTTTTTATTAT | ACTGGGGCTTGTTGTCATTCTG |
