# Supplementary material for: Bacillus subtilis Produces Amino Acids to Stimulate Protein Synthesis in Ruminal Tissue Explants via the Phosphatidylinositol-4,5-Bisphosphate 3-Kinase Catalytic Subunit Beta–Serine/Threonine Kinase–Mammalian Target of Rapamycin Complex 1 Pathway
Source: Front Vet Sci. 2022 Jun 27;9:852321. doi: 10.3389/fvets.2022.852321 (PMC9272757; doi:10.3389/fvets.2022.852321)
Supplement: Supplementary file 3 [file Data_Sheet_3.docx]

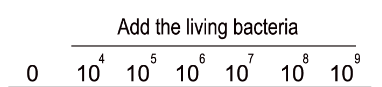
**Figure 5A**


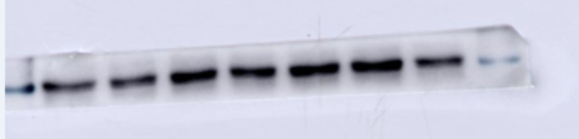


**p-mTOR**


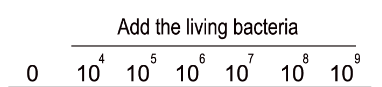


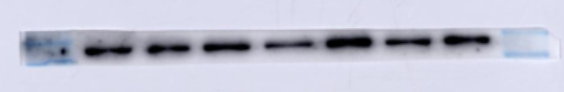


**PIK3CB**


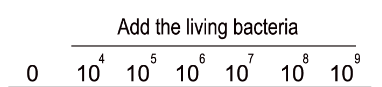


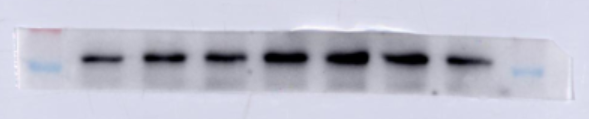


**p-P70S6K**


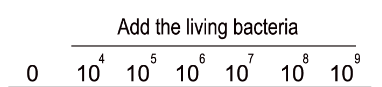


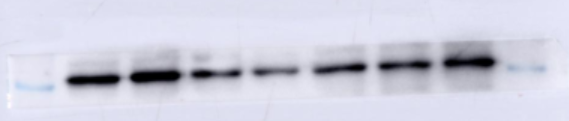


**p-PDCD4**


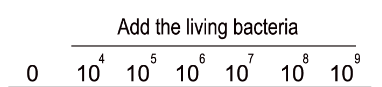


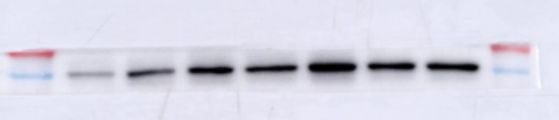


**p-AKT**


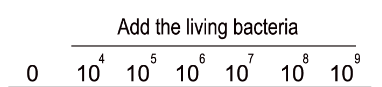


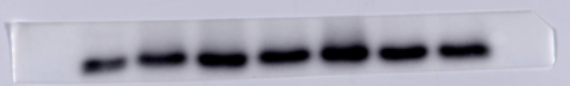


**p-4EBP1**


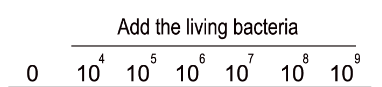


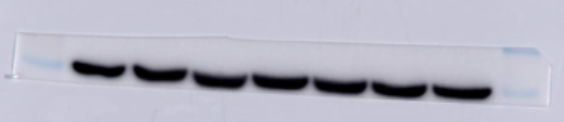


**β-Actin**


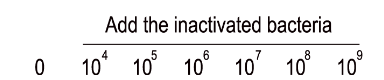
**Figure 5B**


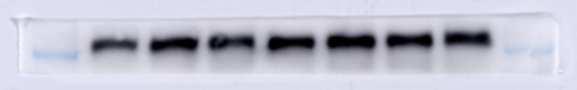


**p-mTOR**


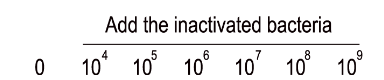


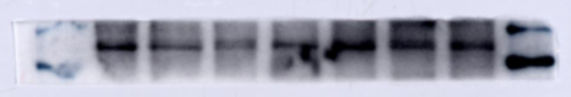


**PIK3CB**


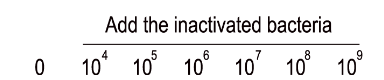


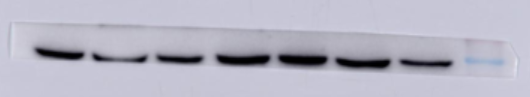


**p-P70S6K**


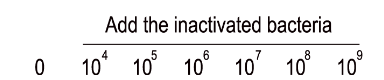


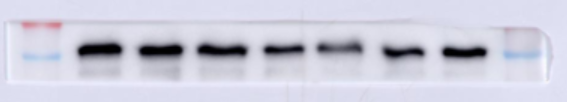


**p-PDCD4**


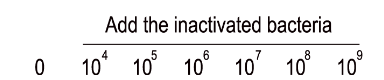


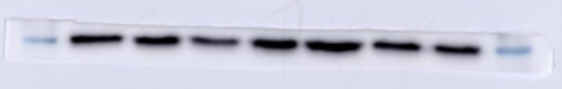


**p-AKT**


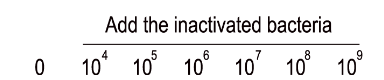


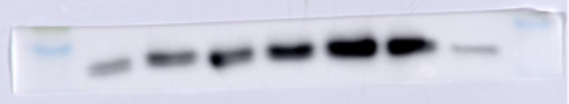


**p-4EBP1**


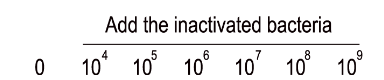


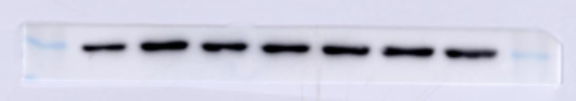


**β-Actin**

**Figure 6**


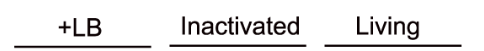


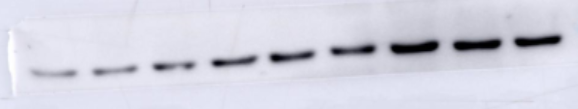


**p-mTOR**


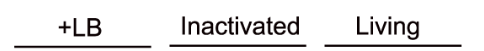


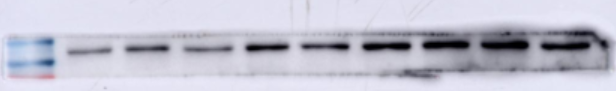


**PIK3CB**


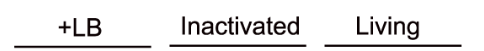


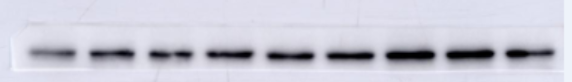


**p-P70S6K**


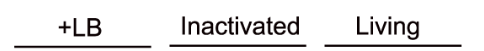


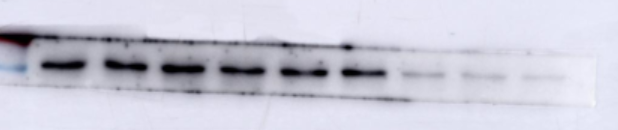


**p-PDCD4**


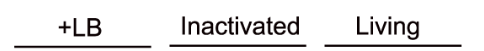


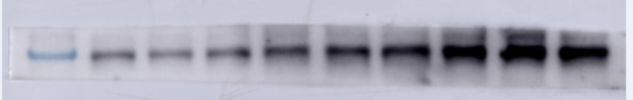


**p-AKT**


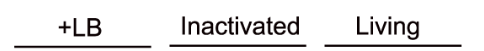


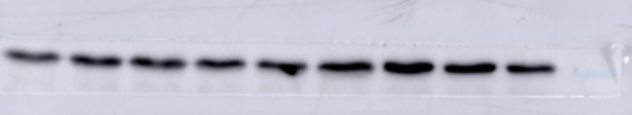


**p-4EBP1**


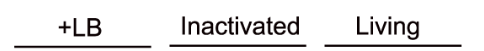


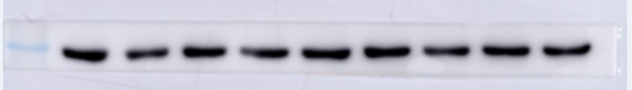


**β-Actin**


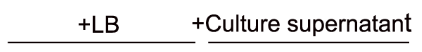
**Figure 8**


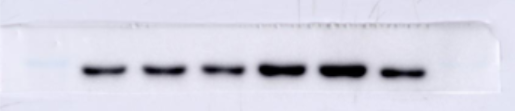


**p-mTOR**


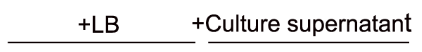


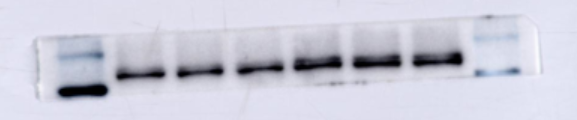


**PIK3CB**


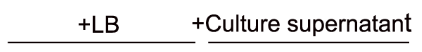


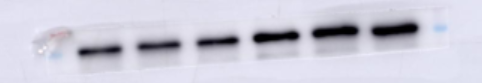


**p-P70S6K**


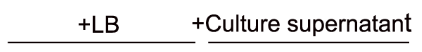


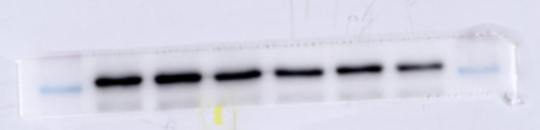


**p-PDCD4**


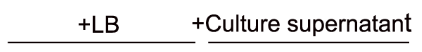


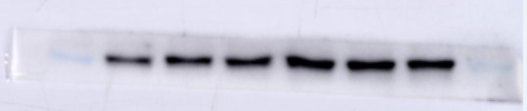


**p-AKT**


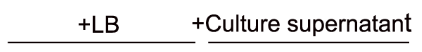


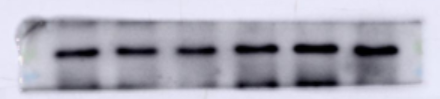


**p-4EBP1**


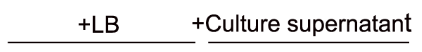


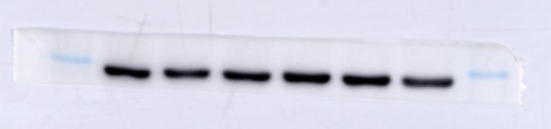


**β-Actin**


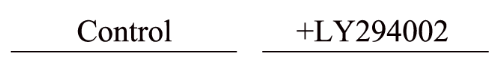
**Figure 9A**


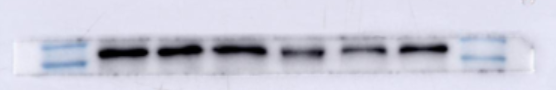


**PIK3CB**


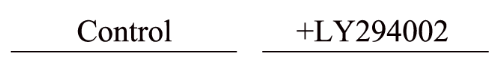


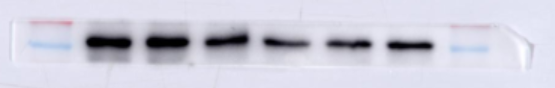


**p-AKT**


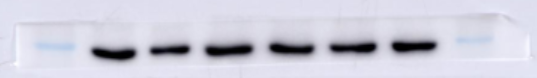

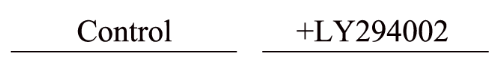


**β-Actin**


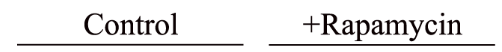


**Figure 9B**


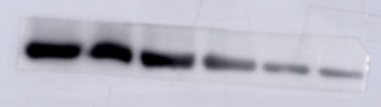


**p-mTOR**


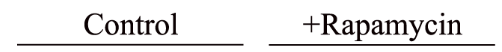


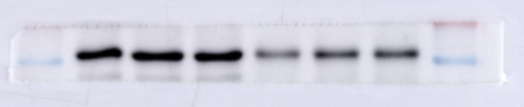


**p-P70S6K**


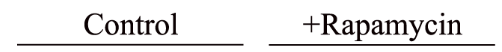


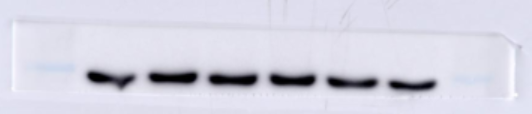


**β-Actin**
